# Human TMEFF1 is a restriction factor for herpes simplex virus in the brain

**DOI:** 10.1038/s41586-024-07745-x

**Published:** 2024-07-24

**Authors:** Yi-Hao Chan, Zhiyong Liu, Paul Bastard, Noopur Khobrekar, Kennen M. Hutchison, Yasuhiro Yamazaki, Qing Fan, Daniela Matuozzo, Oliver Harschnitz, Nacim Kerrouche, Koji Nakajima, Param Amin, Ahmad Yatim, Darawan Rinchai, Jie Chen, Peng Zhang, Gabriele Ciceri, Jia Chen, Kerry Dobbs, Serkan Belkaya, Danyel Lee, Adrian Gervais, Kürşad Aydın, Ayse Kartal, Mary L. Hasek, Shuxiang Zhao, Eduardo Garcia Reino, Yoon Seung Lee, Yoann Seeleuthner, Matthieu Chaldebas, Rasheed Bailey, Catherine Vanhulle, Lazaro Lorenzo, Soraya Boucherit, Flore Rozenberg, Nico Marr, Trine H. Mogensen, Mélodie Aubart, Aurélie Cobat, Olivier Dulac, Melike Emiroglu, Søren R. Paludan, Laurent Abel, Luigi Notarangelo, Richard Longnecker, Greg Smith, Lorenz Studer, Jean-Laurent Casanova, Shen-Ying Zhang

**Affiliations:** 1 St. Giles Laboratory of Human Genetics of Infectious Diseases, Rockefeller Branch, The Rockefeller University, New York, NY USA; 2 Paris Cité University, Imagine Institute, Paris, France; 3 Pediatric Hematology-Immunology and Rheumatology Unit, Necker Hospital for Sick Children, Assistance Publique-Hôpitaux de Paris (AP-HP), Paris, France; 4 Laboratory of Human Genetics of Infectious Diseases, Necker Branch, INSERM U1163, Necker Hospital for Sick Children, Paris, France; 5 The Center for Stem Cell Biology & Developmental Biology Program, Sloan Kettering Institute for Cancer Research, New York, NY USA; 6 Department of Microbiology-Immunology, Northwestern University Feinberg School of Medicine, Chicago, IL USA; 7 Laboratory of Clinical Immunology and Microbiology, National Institute of Allergy and Infectious Diseases, National Institutes of Health, Bethesda, MD USA; 8 Human Technopole, Milan, Italy; 9 Department of Molecular Biology and Genetics, Bilkent University, Ankara, Turkey; 10 Department of Pediatric Neurology, Faculty of Medicine, Istanbul Medipol University, Istanbul, Turkey; 11 Child Neurology Department, Selcuk University, Konya, Turkey; 12 CHU Rouen Normandie, Rouen, France; 13 Laboratory of Virology, Assistance Publique-Hôpitaux de Paris (AP-HP), Cochin Hospital, Paris, France; 14 Research Branch, Sidra Medicine, Doha, Qatar; 15 Department of Biomedicine, Aarhus University, Aarhus, Denmark; 16 Department of Infectious Diseases, Aarhus University Hospital, Aarhus, Denmark; 17 Center for Immunology of Viral Infections, Aarhus University, Aarhus, Denmark; 18 Pediatric Neurology Department, Necker Hospital for Sick Children, Paris-City University, Paris, France; 19 Department of Pediatric Neurology, Necker Hospital for Sick Children, AP-HP, Paris, France; 20 Department of Pediatric Infectious Diseases, Faculty of Medicine, Selcuk University, Konya, Turkey; 21 Department of Rheumatology and Inflammation Research, Institute of Medicine, Sahlgrenska Academy, University of Gothenburg, Göteborg, Sweden; 22 Howard Hughes Medical Institute, New York, NY USA

**Keywords:** Disease genetics, Herpes virus, Restriction factors

## Abstract

Most cases of herpes simplex virus 1 (HSV-1) encephalitis (HSE) remain unexplained^1,2^. Here, we report on two unrelated people who had HSE as children and are homozygous for rare deleterious variants of *TMEFF1*, which encodes a cell membrane protein that is preferentially expressed by brain cortical neurons. TMEFF1 interacts with the cell-surface HSV-1 receptor NECTIN-1, impairing HSV-1 glycoprotein D- and NECTIN-1-mediated fusion of the virus and the cell membrane, blocking viral entry. Genetic TMEFF1 deficiency allows HSV-1 to rapidly enter cortical neurons that are either patient specific or derived from CRISPR–Cas9-engineered human pluripotent stem cells, thereby enhancing HSV-1 translocation to the nucleus and subsequent replication. This cellular phenotype can be rescued by pretreatment with type I interferon (IFN) or the expression of exogenous wild-type *TMEFF1*. Moreover, ectopic expression of full-length TMEFF1 or its amino-terminal extracellular domain, but not its carboxy-terminal intracellular domain, impairs HSV-1 entry into NECTIN-1-expressing cells other than neurons, increasing their resistance to HSV-1 infection. Human TMEFF1 is therefore a host restriction factor for HSV-1 entry into cortical neurons. Its constitutively high abundance in cortical neurons protects these cells from HSV-1 infection, whereas inherited TMEFF1 deficiency renders them susceptible to this virus and can therefore underlie HSE.

## Abstract (web summary)

A study of two childhood cases of herpes simplex encephalitis shows that TMEFF1 interacts with the HSV-1 cell-surface receptor NECTIN-1, preventing HSV-1 from fusing with the cell membrane and entering cortical neurons.

## Main

HSV-1 is an enveloped double-stranded DNA virus that infects people worldwide^3^. After silent or benign infections of oral or nasal epithelial cells, HSV-1 is thought to be transferred to the trigeminal ganglia by retrograde transport from the site of primary infection, establishing latency. Latent infection in the trigeminal ganglia can be reactivated by various triggers, causing herpes labialis, which is also benign. During primary infection (typically in childhood) or reactivation (typically in adulthood), HSV-1 can also cause life-threatening HSE, which has an incidence of about 1–2 per 100,000 individuals per year, corresponding to a prevalence of about 1 per 10,000 inhabitants^3^. The prevalence of HSE peaks in childhood, between the ages of three months and six years^4^. Infection through the trigeminal ganglia typically underlies brainstem encephalitis (around 5% of cases), whereas infection through the olfactory nerve underlies forebrain encephalitis (about 95% of cases)^4,5^. Although rare, HSE is the most common sporadic viral encephalitis in the Western world and perhaps globally. Untreated, it is typically fatal. With acyclovir treatment, which inhibits the synthesis of viral DNA and HSV-1 replication, mortality has fallen to about 20%, but most survivors suffer from neurological sequelae, which are often severe^6^. Remarkably, HSV-1 does not spread to other tissues, even the nasal and oral epithelia, before, during or after HSE^3,4^. Affected patients therefore display a specific vulnerability to HSV-1 in the central nervous system (CNS). This contrasts with the various forms of HSV-1 disease in patients with leukocyte immunodeficiencies, in whom HSV-1 can circulate in the bloodstream and infect various mucocutaneous tissues, but usually not the CNS^7^.

Despite the sporadic nature of HSE, the contrast between the high prevalence of HSV-1 infection and the low prevalence of HSE led to the search for a human monogenic basis for this disease^2^. Single-gene inborn errors of immunity that underlie forebrain HSE have been found in the genes that encode TLR3, UNC93B1, TRIF, TRAF3, TBK1, NEMO and IRF3 in the TLR3-dependent type I IFN-inducing pathway, TFIIIA (GTF3A) in the RIG-I-dependent type I IFN-inducing pathway, and IFNAR1, TYK2, STAT1 and STAT2 in the type I IFN response pathway^2,8–18^. TLR3 is an endosomal sensor of double-stranded RNAs, which may be intermediates or by-products of viral infections. TLR3 also controls the tonic levels of type I interferons, at least in fibroblasts and cortical neurons derived from induced pluripotent stem cells (iPSCs), possibly through unknown endogenous agonists^19^. RIG-I is a cytosolic sensor of double-stranded RNAs^20^. Other forebrain HSE-causing germline mutations have been discovered in *SNORA31* (ref. ^21^) and *RIPK3* (ref. ^22^), whereas mutations in *DBR1* underlie brainstem HSE^23^. The cellular basis of HSE was clarified through studies of iPSC-derived cells in the CNS. Mutations affecting the TLR3-type I IFN pathway impair cortical neuron-intrinsic immunity to HSV-1, whereas trigeminal-ganglia neurons with and without these mutations are equally vulnerable to the virus^24,25^. Mutations of *SNORA31* or *RIPK3* also affect cortical neuron-intrinsic immunity to HSV-1 (ref. ^21,22^). These deficiencies have little impact on leukocytes, which is consistent with the lack of HSV-1 dissemination in patients with HSE. The mechanisms that prevent HSV-1 infection in the brain therefore seem to be intrinsic to brain-resident cells. A genetic aetiology has been established for only 5–10% of children with HSE to date. We hypothesize that other HSE patients without mutations of known HSE-causing genes may carry autosomal recessive inborn errors that disrupt other mechanisms of brain-intrinsic immunity to HSV-1.

## Rare *TMEFF1* variants in two HSE patients

**Fig. 1:**
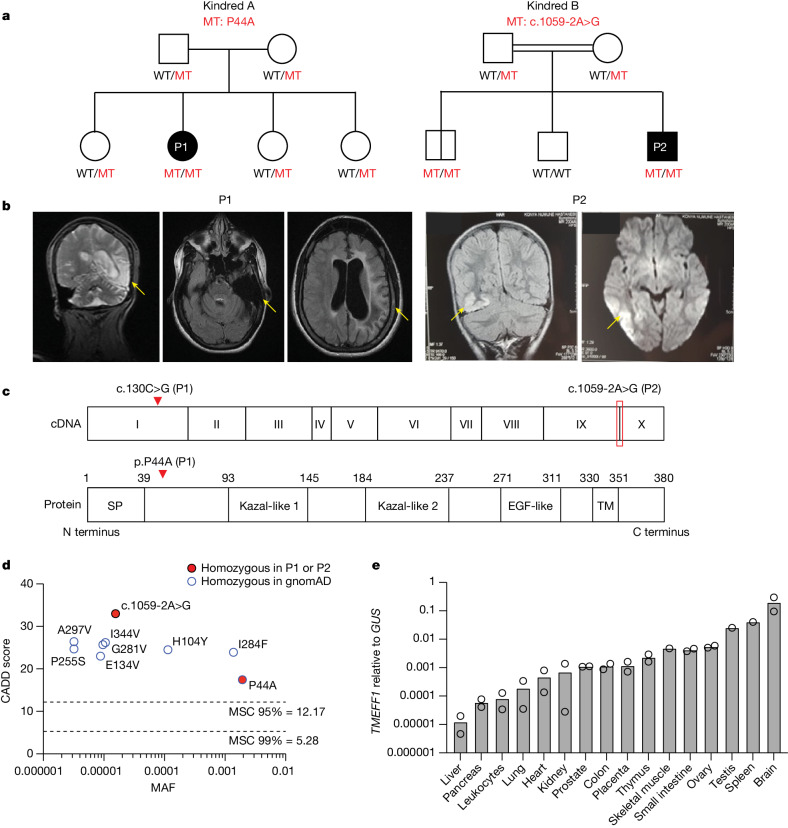
Homozygous *TMEFF1* variants in two unrelated children with HSE. **a**, Family pedigree of patients 1 and 2 (P1 and P2), with segregation of the *TMEFF1* mutations (red). **b**, Brain images for P1 and P2, with yellow arrows showing the lesions observed during HSE. **c**, Schematic of TMEFF1 cDNA and protein structure and the position of the two mutated residues. SP, signal peptide; TM, transmembrane domain. **d**, Graph showing the CADD scores of all *TMEFF1* non-synonymous or essential splice-site variants reported in the homozygous state in the gnomAD database (v.4.1.0.) and their MAFs. Mutation significance cut-offs (MSCs) are shown for 95% and 99% confidence intervals. **e**, Amounts of *TMEFF1* mRNA, as measured by RT–qPCR, in various human tissues. Data shown are from two independent experiments. *GUS*, β-glucuronidase. Source Data

**Extended Data Fig. 1:**
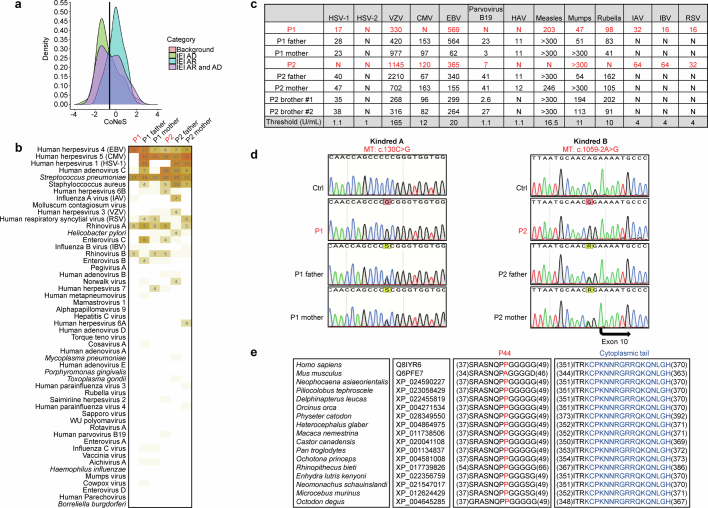
Homozygous *TMEFF1* variants in two unrelated children with HSE. **a**, CoNeS analysis of negative selection for *TMEFF1* plotted against the density distribution for genes underlying autosomal dominant (AD) inborn errors of immunity (IEI), autosomal recessive (AR) IEI, and AR/AD IEI. **b**, VirScan test for antibodies against a wide range of viruses and other pathogens in the serum of the two patients, 8 years (P1) and 10 years (P2) after the HSE episode, and in their parents. The numerical values in the heatmap represent the number of enriched non-overlapping peptides recognized by the antibodies in the serological sample. **c**, Viral serological test on the two patients, 8 years (P1) and 10 years (P2) after the HSE episode, and their relatives. Samples with values below the detection threshold are indicated as N (negative). **d**, Electropherogram showing the *TMEFF1* gDNA sequences surrounding the mutations of interest, in a healthy control (Ctrl), P1, P2, and their parents. **e**, Alignment of TMEFF1 protein sequences around the P44 residue or the cytoplasmic tail, across species. Numbers in parentheses indicate the amino-acid position in each organism.

From our whole-exome sequencing database of 319 HSE patients, we excluded the 30 patients carrying previously reported or unreported deleterious mutations of any of the 15 known HSE-causing genes^1^. We then carried out a genome-wide, unbiased search for new candidate genes under an autosomal recessive inheritance model with genetic homogeneity (genes mutated in at least two patients). We searched for homozygous non-synonymous or splice-site (affecting the essential splicing sites or the intron branchpoints^26^) single-nucleotide variants or copy-number variants with a global minor allele frequency (MAF) of less than 0.01 in the Genome Aggregation Database (gnomAD, v.4.1.0) and a combined annotation-dependent depletion (CADD) score^27^ above the corresponding mutation significance cut-off^28^. We excluded genes with a gene damage index above 13.83, which is used as the cut-off for human genes underlying autosomal recessive life-threatening diseases^29^. We found two unrelated HSE patients homozygous for coding single-nucleotide variants in *TMEFF1*, encoding tomoregulin-1 (a transmembrane protein with epidermal growth factor (EGF)-like and two follistatin-like domains 1, TMEFF1) (Fig. 1a–c). The human *TMEFF1* gene is highly conserved, with a gene damage index of 0.63 and a consensus negative selection score of −0.58 (refs. ^29,30^) (Supplementary Table 1 and Extended Data Fig. 1a), consistent with an autosomal recessive predisposition to life-threatening diseases. There were no patients homozygous for these or for any similarly selected *TMEFF1* variants in 8,500 exomes of a control cohort consisting of patients with other infectious diseases from our in-house whole-exome sequencing database (*P* = 1.6 × 10^−4^). The p.Pro44Ala (p.P44A) variant (MAF = 0.0019) in patient 1 (P1) was present in the homozygous state in two individuals, whereas the c.1059-2A>G variant of patient 2 (P2) (MAF = 0.0000156) was not present in the homozygous state in the gnomAD database (v.4.1.0), which contains whole-exome sequence or whole-genome sequence data from 807,162 individuals representative of the general population. Apart from the two homozygous carriers of Pro44Ala, only 11 of 807,162 individuals from the gnomAD database were homozygous for seven other similarly selected variants (His104Tyr (H104Y), Glu134Val (E134V), Pro255Ser (P255S), Gly281Val (G281V), Ile284Phe (I284F), Ala297Val (A297V) and Ile344Val (I344V)) of *TMEFF1*, resulting in a cumulative frequency of homozygotes of 1.4 × 10^−5^ (confidence interval: 5.6 × 10^−6^–2.2 × 10^−5^) in gnomAD. There is, therefore, an enrichment in homozygous non-synonymous *TMEFF1* variants in the HSE cohort relative to the gnomAD database representative of the general population (*P* = 10^−5^). Finally, there were no homozygous carriers of any other more common coding non-synonymous or splice variants of *TMEFF1* in gnomAD, and the cumulative MAF of predicted loss-of-function variants was only 0.0001 (all carriers being heterozygous, with no homozygous carriers reported) (Fig. 1d). Since the first identification of its orthologue Tmeff1 in *Xenopus laevis* in 1996 (ref. ^31^), the function of the TMEFF1 protein in animals and humans has remained unclear. There is no evidence to indicate that TMEFF1 is involved in antiviral immunity. However, in *Xenopus*, as in mice, the expression and function of Tmeff1 seem to be specific to the CNS^31–33^. Likewise, the human TMEFF1 protein and mRNA are most abundant, and almost exclusively present, in the brain (https://www.gtexportal.org, https://proteinatlas.org and Fig. 1e). We therefore hypothesized that an autosomal recessive deficiency of TMEFF1 might underlie HSE in both patients through the disruption of brain-intrinsic immunity.

## *TMEFF1* is an HSE candidate gene

P1 is a 19-year-old woman born to non-consanguineous parents of Algerian and Moroccan origin living in France (Fig. 1a). She had a severe course of HSE at two-and-a-half years of age (Fig. 1b), with large cortical lesions in the temporal region. P2 is a 19-year-old man born to consanguineous parents of Turkish origin living in Turkey (Fig. 1a). He had encephalitis at the age of five years with large cortical lesions in the temporo-occipital regions; he was hospitalized and diagnosed with HSE (Fig. 1b). Neither patient had any other remarkable prior or subsequent medical history, despite having been infected with many common viruses, as demonstrated by conventional serological testing and VirScan^34^ (Extended Data Fig. 1b,c). They were both treated with intravenous acyclovir, and both recovered but had severe neurological sequelae. Sanger sequencing confirmed that P1 was homozygous for a missense variant (c.130C>G, p.P44A) and that P2 was homozygous for an essential splice-site variant (c.1059-2A>G) (Extended Data Fig. 1d). Familial segregation of the variants confirmed autosomal recessive inheritance, with the parents of both patients being heterozygous for the corresponding variant (Fig. 1a and Extended Data Fig. 1d). P1’s three siblings were also heterozygous for the missense variant. One of P2’s siblings was homozygous wild-type (WT) and another had a history of benign HSV-1 infection, as confirmed by serological testing, and was homozygous for the same variant as P2, indicating incomplete clinical penetrance for HSE. Incomplete clinical penetrance has also been reported for other genetic aetiologies and is consistent with the typically sporadic nature of HSE^1^. No remarkable medical history was reported for any of the relatives of either of the patients. Human TMEFF1 is a transmembrane protein expressed on the cell surface^31–33,35^. It consists of a large N-terminal extracellular segment containing two Kazal-like domains and one EGF-like domain, a transmembrane domain and a short C-terminal intracellular segment (Fig. 1c). P1’s missense variant affects a highly conserved residue at the N terminus of the protein (CADD 17.44), whereas P2’s variant affects an essential splicing variant and is predicted to lead to abnormal splicing of the highly conserved cytoplasmic C-terminal tail of the protein (CADD 33) (Extended Data Fig. 1e). Both variants are therefore probably deleterious and possibly pathogenic. Moreover, no other candidate genes were identified among the 11 and 29 biallelic rare non-synonymous variants at other loci found in P1 and P2, respectively, based on their expression or function, and only two variants in each patient (differing between the patients) were predicted to be loss-of-function (Supplementary Table 1). Overall, *TMEFF1* was the most plausible candidate gene underlying HSE in these two patients.

## Subcellular distribution of TMEFF1

**Fig. 2:**
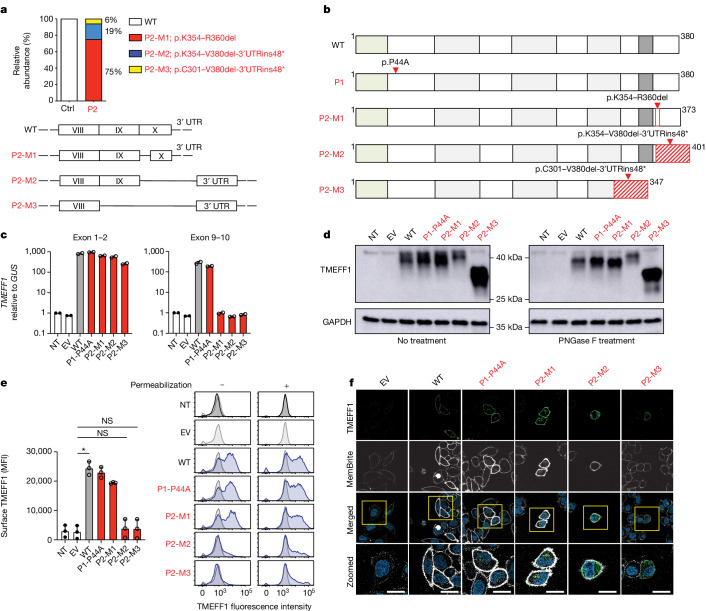
Expression and subcellular distribution of mutant TMEFF1 proteins in vitro. **a**, Relative abundance of *TMEFF1* cDNA isoforms generated from mRNA extracted from primary fibroblasts from a healthy control (Ctrl) and P2, as assessed by TOPO-TA cloning. Mutant isoforms are shown in red. **b**, Schematic representation of TMEFF1 protein structure and the impact of P1’s missense mutation, and P2’s mutation resulting in three mutant isoforms (P2-M1, P2-M2, P2-M3). **c**, Amounts of *TMEFF1* mRNA, as measured by RT–qPCR on HEK293T cells, not transfected (NT) or transfected with an empty vector (EV) or with plasmids containing WT or various patient-specific mutant *TMEFF1* cDNA sequences. Two probes, targeting exons 1–2 (left) and exons 9–10 (right) of *TMEFF1*, were used. Data are presented as mean ± s.d. **d**, TMEFF1 protein levels, as assessed by western blotting on HEK293T cells, NT or transfected with various plasmids as in **c**. Protein lysates were either left untreated or were treated with peptide:*N*-glycosidase F (PNGase F). GAPDH, glyceraldehyde-3-phosphate dehydrogenase. **e**, TMEFF1 protein levels, as assessed by flow cytometry, in permeabilized and unpermeabilized HEK293T cells (right), NT or transfected with various plasmids as in **c**. Cell-surface TMEFF1 expression was quantified in unpermeabilized cells (left). Data are presented as mean ± s.d. Statistical analysis was done with Kruskal–Wallis tests with Dunn’s test for multiple comparisons. NS, not significant; *P < 0.05. MFI, mean fluorescence intensity. **f**, TMEFF1 immunostaining in HeLa cells transfected with an EV or with plasmids containing WT or various patient-specific mutant *TMEFF1* cDNA sequences. MemBrite is a cell membrane marker. Blue indicates DAPI chromosome staining. Scale bars, 20 μm. The data shown in **c**–**f** are representative of three independent experiments. Source Data

**Extended Data Fig. 2:**
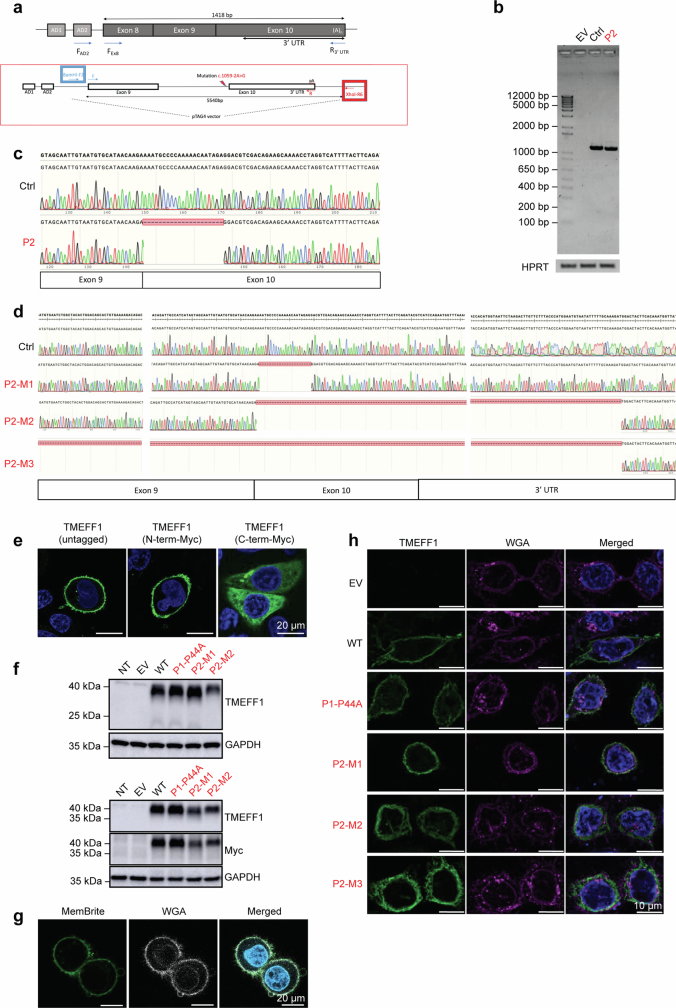
Assessment of the impact of P2’s variant, and the subcellular distribution of TMEFF1. **a**, Schematic representation of the experimental design of the TOPO-cloning (upper panel) and exon-trapping (lower panel) experiments. In brief, for TOPO-TA, 1418 bp of cDNA, corresponding to exons 8 to 10 and including the 3’UTR and a polyA tail, for a control or P2 was extracted, amplified and inserted into a reporter vector for cDNA sequencing. For the exon-trapping experiment, a 5540 bp gDNA sequence for a control or P2 was amplified with forward and reverse primers, as shown. All of exons 9 and 10, including most of the 3’UTR, was amplified. The amplified gDNA was then digested with the BamHI and Xho1 enzymes and inserted into a pTAG4 vector, which was then used to transfect COS-7 cells for DNA extraction. P2’s essential splice-site mutation is indicated by a lightning bolt. **b**, Image of the gel, showing the fragment of cDNA amplified in the exon-trapping experiment, after amplification, insertion into the pTAG4 plasmid, the transfection of COS-7 cells, extraction of mRNA and amplification of the cDNA by PCR. The fragment obtained for the patient is of slightly lower molecular weight. Representative data from three independent experiments are shown. **c**, Sequencing results for the cDNA extracted from COS-7 cells following transfection with the pTAG4 plasmid containing gDNA from a control and P2, after the exon-trapping experiment as described in **a**-**b**. Experiments with the patient’s cDNA indicated that a single transcript lacking the first 21 nucleotides of exon 10 was produced. The data shown are representative of three independent experiments. **d**, Sequencing results for the *TMEFF1* cDNA extracted from primary fibroblasts from a control and P2, after the TOPO-TA experiment. Experiments with the patient’s cDNA indicated that a single transcript lacking the first 21 nucleotides of exon 10 (P2-M1) was produced, as in the exon-trapping experiment, but there were also two additional transcripts with larger deletions encompassing the entire coding sequence of exon 10 and part of the 3’UTR (P2-M2) and encompassing the entire coding sequence of exons 9 and 10 and part of the 3’UTR (P2-M3). Data representative of three independent experiments are shown. **e**, Immunostaining for TMEFF1 in HeLa cells transfected with plasmids containing wild-type (WT) untagged, or N-ter Myc-tagged or C-ter Myc-tagged *TMEFF1* cDNA sequences, showing that the C-ter Myc-tag impairs the expression of TMEFF1 at the cell surface. The data shown are representative of three independent experiments. **f**, Immunoblotting for TMEFF1 in HEK293T cells transfected with plasmids containing wild-type (WT) or mutant TMEFF1 sequences without a tag (upper panel), or with a C-ter Myc-tag (lower panel). The data shown are representative of three independent experiments. **g**, Cell membrane labeling with two different staining kits, MemBrite (green) and wheatgerm agglutinin (WGA, white). **h**, TMEFF1 immunostaining in HeLa cells transfected with an empty vector (EV) or with plasmids containing wild-type (WT) or various patient-specific mutant *TMEFF1* cDNA sequences. WGA: cell membrane marker. Blue indicates DAPI chromosome staining. Data representative of three independent experiments are shown.

We investigated the expression of the mutant (MT) TMEFF1 proteins of the two patients by plasmid-mediated cDNA overexpression in vitro. We first assessed the impact of the essential splice-site variant of P2 on mRNA production. We performed exon trapping with P2’s genomic DNA (Extended Data Fig. 2a–c) and topoisomerase-based TA (TOPO-TA) cloning with cDNA from P2’s fibroblasts (Fig. 2a). We found that the c.1059-2A>G essential splice-site variant led to abnormal splicing of *TMEFF1* cDNA, resulting in three different abnormal transcripts, with different predicted impacts on protein levels (Fig. 2a,b and Extended Data Fig. 2d). The most abundant (75%) transcript included a deletion of 21 nucleotides (P2-M1, p.Lys354–Arg360del (p.K354–R360del)), whereas the other two had a deletion of exon 10 and an insertion of part of the 3′-untranslated region (UTR) (P2-M2, p.Lys354–Val380del-3′UTRins48* (p.K354–V380del-3’UTRins48*), 19%), or a deletion of exons 9 and 10 along with the same insertion of part of the 3′-UTR (P2-M3, p.Cys301–Val380del-3′UTRins48* (p.C301–V380del-3′UTRins48*), 6%). We then studied the expression and subcellular distribution of the MT proteins of P1 and P2 by overexpression in HEK293T and HeLa cells, respectively. After the transient transfection of HEK293T cells with plasmids containing WT or MT *TMEFF1* cDNA, similar levels of *TMEFF1* mRNA were detected by quantitative PCR with reverse transcription (RT–qPCR) with a probe spanning the N-terminal domain of TMEFF1 (Fig. 2c). However, with a probe spanning the C-terminal domain of *TMEFF1*, similar levels of *TMEFF1* mRNA were detected for P1’s MT and the WT, whereas P2-M1, P2-M2 and P2-M3 were undetected. Western-blot analyses of extracts of these cells with an antibody specific for the N-terminal region of TMEFF1 revealed that P1’s P44A protein had a similar molecular weight to the WT protein, whereas P2’s M1 and M3 proteins were smaller than the WT protein and the P2-M2 protein was larger than the WT protein (Fig. 2d). All but one of the MT proteins were produced in similar amounts to the WT TMEFF1, the exception being P2-M2, which was produced in smaller, but nevertheless detectable, amounts. All WT and MT TMEFF1 proteins were glycosylated; treatment of the cell lysates with peptide *N*-glycosidase F before western blotting removed the upper band and left only one band detected by the anti-TMEFF1 antibody. The cell-surface expression of the WT and MT TMEFF1 proteins was analysed by fluorescence-activated cell sorting and confocal microscopy in transfected HeLa cells. Consistent with previously reported data showing that human TMEFF1 is a transmembrane protein expressed on the cell surface^31–33,35^, plasmid transfection led to WT TMEFF1 expression on the cell membrane when an untagged plasmid was used, whereas this protein was located mostly in the cytoplasm when a C-terminally tagged plasmid was used (Extended Data Fig. 2e,f). In the overexpression system using untagged TMEFF1 vectors, the P44A and P2-M1 TMEFF1 proteins were expressed on the cell membrane, as was WT TMEFF1, whereas P2-M2 was found both on the cell membrane and in the cytoplasm, and P2-M3 was present solely in the cytoplasm (Fig. 2e,f and Extended Data Fig. 2g,h). The functions of the M2 and M3 TMEFF1 proteins of P2 may therefore be affected by the weak or abolished expression, respectively, of these proteins at the cell membrane, whereas the functions of P1’s P44A protein and P2’s M1 TMEFF1 protein may be affected by other mechanisms.

## HSV-1 susceptibility in neurons

**Fig. 3:**
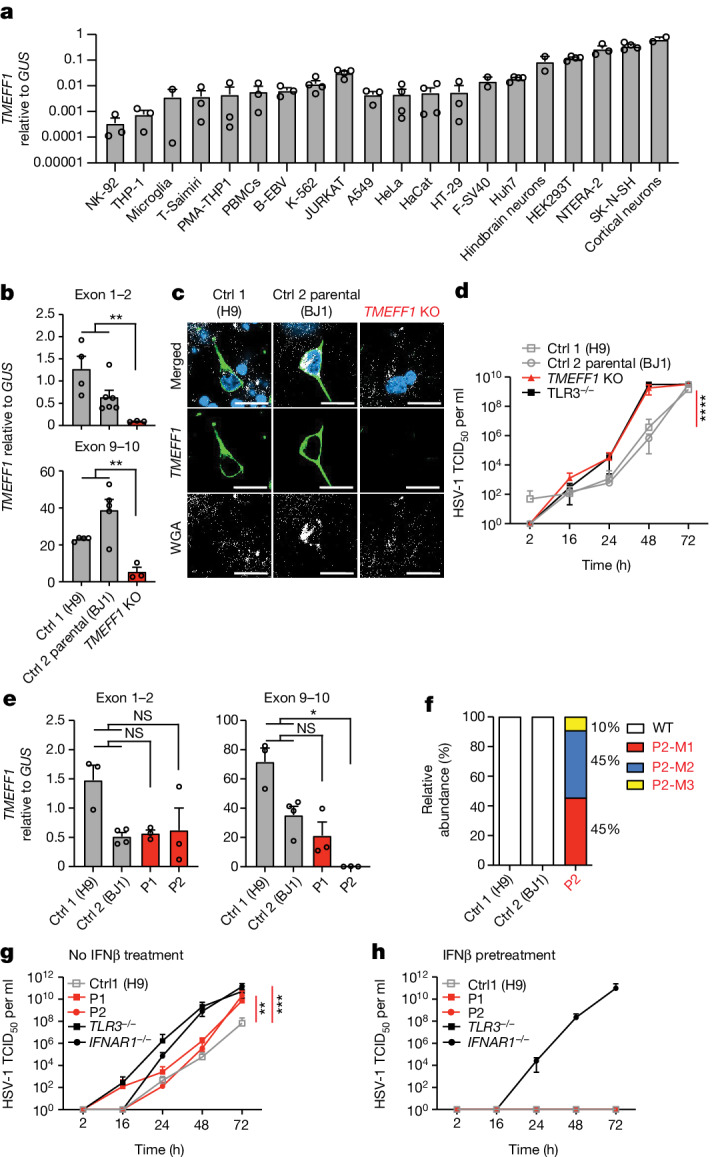
Enhanced HSV-1 susceptibility in TMEFF1-deficient hPSC-derived cortical neurons. **a**, Levels of *TMEFF1* mRNA, as determined by RT–qPCR, in various human cell lines or primary cells. **b**, *TMEFF1* mRNA levels were determined by RT–qPCR in cortical neurons from control and *TMEFF1*-KO hPSCs. **c**, TMEFF1 protein expression was studied by confocal microscopy on cortical neurons derived from healthy control and *TMEFF1*-KO hPSCs. Cells were fixed and stained for TMEFF1 (anti-TMEFF1 antibody, green), cell membrane (wheat germ agglutinin (WGA), white) and chromosomes (DAPI, blue). Scale bar, 10 μm. **d**, Cortical neurons derived from hPSCs from healthy controls, *TMEFF1*-KO hPSCs and *TLR3*^−/−^ hPSCs were infected with HSV-1 (MOI 0.001) and assessed for HSV-1 titres at the timepoints indicated. TCID_50_, 50% tissue culture infectious dose. **e**, *TMEFF1* mRNA levels were determined by RT–qPCR on hPSC-derived cortical neurons for healthy controls and the two patients with *TMEFF1* mutations (P1 and P2). **f**, Relative abundance of *TMEFF1* cDNA isoforms generated from mRNA extracted from hPSC-derived cortical neurons for healthy controls and P2, as assessed by TOPO-TA cloning. **g**,**h**, hPSC-derived cortical neurons from a healthy control (H9), the patients with *TMEFF1* mutations (P1 and P2) and other *TLR3*^−/−^ and *IFNAR1*^−/−^ HSE patients were infected with HSV-1 (MOI 0.001) and assessed for HSV-1 titres at the timepoints indicated, without (**g**) or with (**h**) IFNβ pretreatment for 18 h. The data shown in **a**, **b**, **d**, **e**, **g** and **h** are mean ± s.e.m. of three independent experiments. Statistical analysis: for **b** and **e**, two-tailed Mann-Whitney *U*-tests; for **d**, **g** and **h**, mean log-transformed relative values were compared between control cells and *TMEFF1*-mutated cells in one-way analysis of variance (ANOVA) with Tukey tests for multiple comparisons. *P < 0.05; **P < 0.01; ***P < 0.001; ****P < 0.0001. Source Data

**Extended Data Fig. 3:**
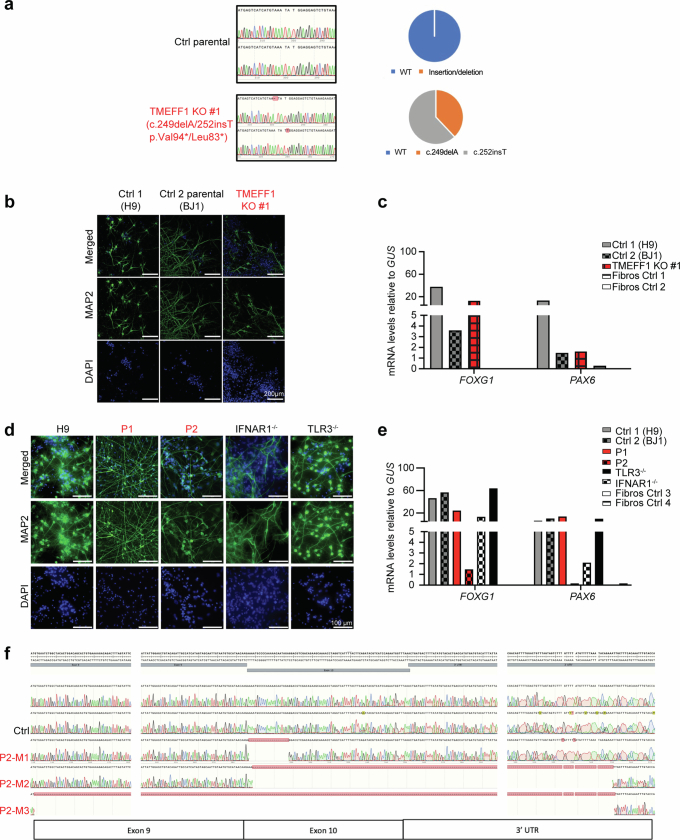
Characterization of hPSC-derived CNS cortical neurons. **a**, Electropherogram representation (left panels) of the CRISPR-Cas9-introduced compound-heterozygous *TMEFF1* mutations confirmed by Sanger sequencing on genomic DNA from a gene-edited TMEFF1 KO hPSC line (TMEFF1 KO #1). Sequencing results for the parental line (Ctrl parental, BJ1) are also shown. The relative abundance of WT or mutated *TMEFF1* cDNA generated from mRNA extracted from the control parental and TMEFF1 KO hPSCs was assessed by TOPO-TA cloning and is shown in the right panels. **b**, Representative images of cortical neurons from controls (Ctrl 1-H9, Ctrl 2 parental-BJ1) and TMEFF1 KO hPSCs. Cells were fixed and stained with DAPI (blue) and for a neuron-specific marker, microtubule-associated protein 2 (MAP2, green). **c**, *FOXG1* and *PAX6* mRNA levels, as measured by RT-qPCR, in cortical neurons from control and TMEFF1 KO hPSCs. SV40-transformed fibroblasts from healthy controls (Fibros ctrl 1, Fibros ctrl 2) were used as a negative control in this assay. **d**, Representative images of cortical neurons from control (H9) and various patient-specific hPSC lines (P1, P2, IFNAR1^−/−^, TLR3^−/−^). Cells were fixed and stained with DAPI (blue), and for a neuron-specific marker MAP2 (green). **e**, *FOXG1* and *PAX6* mRNA levels were measured by RT-qPCR in cortical neurons derived from control and patient-specific hPSC lines. SV40-transformed fibroblasts from healthy controls (Fibros ctrl 3, Fibros ctrl 4) were used as a negative control in this assay. **f**, Electropherogram representation of the three *TMEFF1* mutant isoforms, as detected by TOPO-cloning of cDNA from P2’s hPSC-derived cortical neurons. Source Data

We hypothesized that TMEFF1 acts as a restriction factor for HSV-1 in cortical neurons. Consistent with this hypothesis, *TMEFF1* mRNA levels in human pluripotent stem cell (hPSC)-derived cortical neurons were the highest of all the primary cells and cell lines from the brain and other tissues tested (Fig. 3a). We generated *TMEFF1-*knockout (KO) hPSCs by CRISPR–Cas9 gene-editing^36^, and *TMEFF1*-KO and parental WT hPSCs were then differentiated into cortical neurons^21,37^ (Fig. 3b,c and Extended Data Fig. 3a–c). *TMEFF1* mRNA levels were lower in *TMEFF1*-KO neurons than in WT parental cells (Fig. 3b). Furthermore, no TMEFF1 protein was detected on the cell membrane of *TMEFF1*-KO neurons, whereas TMEFF1 was detected on the cell surface of WT parental neurons (Fig. 3c). After infection with HSV-1 at a multiplicity of infection (MOI) of 0.001, as in hSPC-derived cortical neurons with complete TLR3 deficiency (*TLR3*^−/−^) from a previously reported HSE patient^24^, viral replication rates in *TMEFF1*-KO neurons at various time points were higher than those in neurons differentiated from parental WT hPSCs and hPSCs from another healthy control (Fig. 3d). We then derived cortical neurons from the hPSCs of the two patients and assessed HSV-1 replication in these cells (Fig. 3e–h and Extended Data Fig. 3d,e). *TMEFF1* mRNA levels in the cortical neurons of P1 and P2 were within the range for healthy control cortical neurons when measured with a probe spanning the N-terminal domain of TMEFF1, but these mRNAs were undetectable in cortical neurons from P2 when measured with a probe spanning the C-terminal domain of TMEFF1, consistent with the abnormally spliced mutant transcripts of P2 (Fig. 3e). TOPO-TA cloning of the *TMEFF1* cDNA from P2’s neurons confirmed the impact of the essential splice-site variant, with the detection of the same three MT transcripts previously identified in the patient’s fibroblasts (Fig. 3f and Extended Data Fig. 3f). Nevertheless, the proportions of the three MT transcripts in neurons differed slightly from those in the patient’s fibroblasts, with around 45%, 45% and 10% of the transcripts corresponding to P2-M1, P2-M2 and P2-M3, respectively (Fig. 3f). As in *TMEFF1*-KO cortical neurons, high levels of viral replication were detected in the *TMEFF1*-mutated neurons of the two patients at various time points after infection with HSV-1 at an MOI of 0.001 (Fig. 3g). Viral replication rates were similar to those in TLR3- and IFNAR1-deficient neurons, but higher than those in healthy control neurons (Fig. 3g). This HSV-1 replication phenotype was rescued by IFNβ pretreatment, which can result in the strong induction of antiviral interferon-stimulated genes (ISGs)^38^, in both TMEFF1- and TLR3-deficient neurons, but not in IFNAR1-deficient neurons (Fig. 3h), indicating that TMEFF1 does not control HSV-1 infection through a mechanism affecting the type I IFN response pathway.

## Intact TLR3 and type I IFN responses

**Extended Data Fig. 4:**
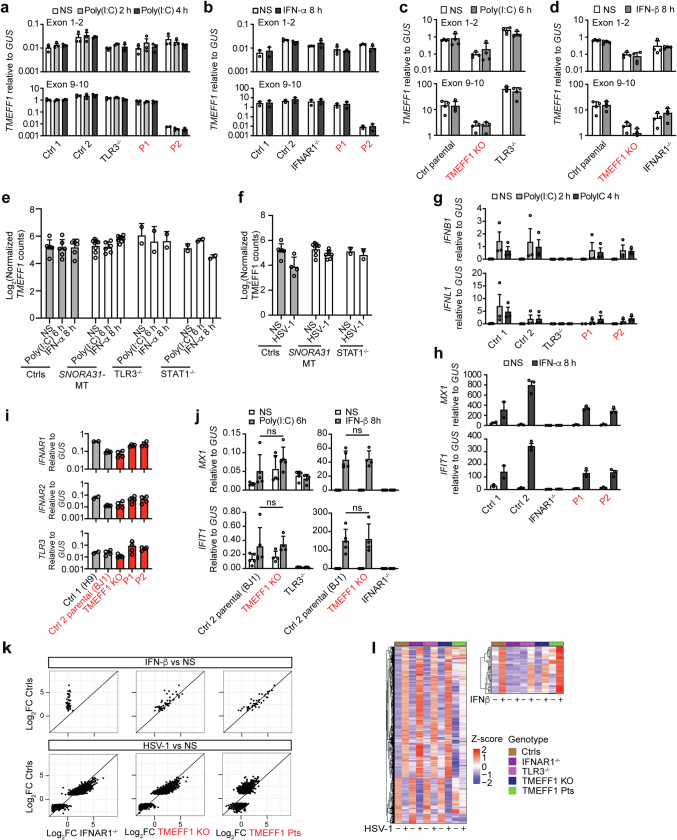
Intact TLR3 and type I IFN responses in *TMEFF1*-mutated cells. **a**, *TMEFF1* mRNA levels were determined by RT-qPCR, in SV40-transformed fibroblasts from the patients with *TMEFF1* mutations, a TLR3^−/−^ HSE patient, and healthy controls treated with poly(I:C) for 2 or 4 h or left untreated (NS). **b**, *TMEFF1* mRNA levels were measured by RT-qPCR, in SV40-transformed fibroblasts from patients with *TMEFF1* mutations, an IFNAR1^−/−^ HSE patient, and healthy controls treated with IFN-α2b for 8 h or left untreated. **c**, *TMEFF1* mRNA levels were measured by RT-qPCR in cortical neurons derived from control parental or TMEFF1 KO hPSCs, and hPSCs from a TLR3^−/−^ HSE patient, after treatment with poly(I:C) for 6 h, or without treatment. **d**, *TMEFF1* mRNA levels were measured by RT-qPCR, in cortical neurons derived from control parental or TMEFF1 KO hPSCs, and hPSCs from an IFNAR1^−/−^ HSE patient treated with IFN-β for 8 h or left untreated. In **a**-**d**, two probes, targeting exons 1-2 (upper panels) and exons 9-10 (lower panels) of *TMEFF1* were used. The data shown are the means ± SEM from three (**a**,**b**) or two (**c**,**d**) independent experiments. **e**, Abundance of *TMEFF1* mRNA, as assessed by RNAseq, in healthy control neurons (Ctrls, *n* = 6), *SNORA31*-mutated (*SNORA31*-MT, *n* = 8), TLR3^−/−^ (*n* = 2) or STAT1^−/−^ (*n* = 2) hPSC-derived cortical neurons treated with poly(I:C) or IFN-α2b, or left unstimulated (NS). Data are presented as mean ± SD. **f**, Abundance of *TMEFF1* mRNA, as assessed by RNAseq, in healthy controls (Ctrls, *n* = 6), *SNORA31*-mutated (*SNORA31*-MT, *n* = 8) or STAT1^−/−^ (*n* = 2) hPSC-derived cortical neurons infected with HSV-1 for 24 h, or left unstimulated (NS). **g**, *IFNB1* (upper panel) or *IFNL1* (lower panel) mRNA levels were measured by RT-qPCR, in SV40-transformed fibroblasts from the patients with *TMEFF1* mutations, a TLR3^−/−^ HSE patient, and healthy controls, after treatment with poly(I:C) for 2 or 4 h or without treatment. **h**, *MX1* (upper panel) or *IFIT1* (lower panel) mRNA levels were measured by RT-qPCR, in SV40-transformed fibroblasts from the patients with *TMEFF1* mutations, an IFNAR1^−/−^ HSE patient, and healthy controls, after treatment with IFN-α2b for 8 h, or without treatment. The data shown in **g**, and **h** are the means ± SEM from three independent experiments. **i**, Basal levels of *IFNAR1* (top panel), *IFNAR2* (middle panel), and *TLR3* (lower panel) mRNA were measured by RT-qPCR, in hPSC-derived cortical neurons from healthy controls (Ctrl 1-H9, Ctrl 2-Parental BJ1), TMEFF1 KO hPSCs, or hPSCs from TMEFF1-mutated patients. **j**, Levels of *MX1* (upper panels) or *IFIT1* (lower panels) mRNA were measured by RT-qPCR, in cortical neurons derived from control parental or TMEFF1 KO hPSCs, hPSCs from a TLR3^−/−^ HSE patient, and an IFNAR1^−/−^ HSE patient, with and without treatment with poly(I:C) for 6 h (left panels), or with IFN-β for 8 h (right panels). Statistical analysis was performed with two-tailed Mann-Whitney *U* tests. ns: not significant. **k**, Scatterplots of the mean log_2_ fold-changes in RNAseq-quantified gene induction following stimulation with 100 IU/ml IFN-β for 8 h (upper panel) or HSV-1 (MOI 1) for 24 h (lower panel) in hPSC-derived CNS cortical neurons from two healthy controls (Ctrl1-H9, Ctrl2 parental-BJ1), TMEFF1-mutated patients (TMEFF1 Pts) or TMEFF1 KO hPSCS, or hPSCs from an IFNAR1^−/−^ HSE patient. Each point represents a single gene. Genes with an absolute fold-change in expression > 2 in response to IFN-β or HSV-1 treatment relative to NS samples in the Ctrl group are plotted. **l**, Heatmaps of RNA-Seq-quantified gene expression (z-score-scaled DESeq2 vst-normalization) in hPSC-derived CNS cortical neurons from healthy controls (Ctrl 1-H9, Ctrl 2-Parental BJ1) or TMEFF1 KO hPSCS, or hPSCs from an IFNAR1^−/−^ HSE patient, a TLR3^−/−^ HSE patient and TMEFF1-mutated P1 and P2 (TMEFF1 Pts), not stimulated (NS), stimulated with HSV-1 for 24 h, or stimulated with IFN-β for 8 h. Duplicates were studied for each set of conditions and mean gene expression levels were used for subsequent analyses. The heatmap includes genes with a relative fold-change in expression > 2 in response to HSV-1 or IFN-β treatment relative to NS samples in the Ctrl group. Source Data

We investigated whether TMEFF1 controlled HSV-1 infection in neurons through known TLR3- and type I IFN-mediated or snoRNA31-dependent mechanisms by first determining whether *TMEFF1* was a TLR3- or IFN-inducible gene (an ISG) and whether its expression was regulated by snoRNA31. RT–qPCR showed that *TMEFF1* mRNA levels were not increased by stimulation with poly(I:C) or IFNα in dermal fibroblasts from healthy controls or from the two patients (Extended Data Fig. 4a,b), or by stimulation with poly(I:C) or IFNβ in *TMEFF1* WT control parental or *TMEFF1*-KO hPSC-derived cortical neurons (Extended Data Fig. 4c,d). RNA sequencing (RNA-seq) showed that *TMEFF1* mRNA levels were also similar in cortical neurons with *SNORA31* mutations, in TLR3- or STAT1-deficient neurons and in control neurons (Extended Data Fig. 4e). Moreover, *TMEFF1* mRNA levels were not modulated by stimulation with poly(I:C), IFNα or HSV-1 in hPSC-derived cortical neurons (Extended Data Fig. 4e,f). We then investigated whether TMEFF1 controlled cellular responses to TLR3 or type I IFN. The fibroblasts of the two patients responded normally to extracellular poly(I:C) added for TLR3 stimulation, and to the stimulation of IFNAR1/IFNAR2 with IFNα, in terms of mRNA induction for *IFNB1*, *IFNL1*, *MX1* and *IFIT1* (Extended Data Fig. 4g,h). Moreover, *TMEFF1*-KO and cortical neurons derived from hPSCs from the two patients had normal levels of *TLR3* and *IFNAR1*/*IFNAR2* mRNAs (Extended Data Fig. 4i) and normal responses to TLR3 or IFNAR1/IFNAR2 stimulation in terms of the induction of mRNA for the ISGs tested, including *MX1* and *IFIT1* (Extended Data Fig. 4j). Furthermore, bulk RNA-seq on cortical neurons derived from hPSCs from the two patients, *TMEFF1*-KO and parental WT hPSCs showed that TMEFF1 deficiency did not alter transcriptomic responses to IFNβ after 8 h of stimulation, or those to HSV-1 after 24 h of infection (Extended Data Fig. 4k,l). These data indicate that the mechanism by which TMEFF1 controls HSV-1 infection in cortical neurons is independent of the known TLR3-mediated and type I IFN-mediated antiviral pathways, and independent of snoRNA31. Pretreatment with IFNβ rendered TMEFF1-deficient neurons resistant to HSV-1 infection, but this probably involved compensation resulting from the upregulation of antiviral ISGs. Overall, TMEFF1 controls HSV-1 replication in cortical neurons by mechanisms other than the previously documented neuron-intrinsic antiviral pathways mediated by TLR3, IFNAR1/IFNAR2 and snoRNA31 (refs. ^1,21,39^).

## TMEFF1 restricts HSV-1 infection

**Fig. 4:**
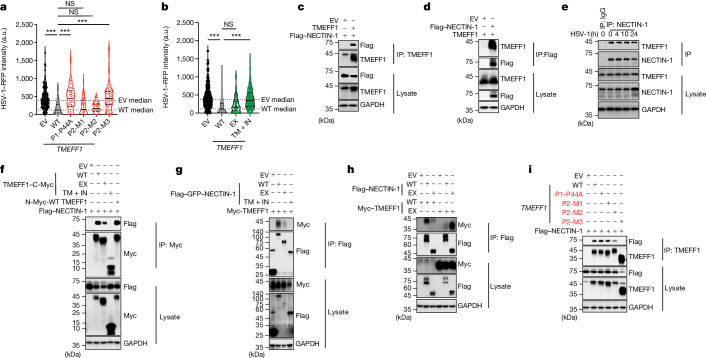
TMEFF1 interacts with NECTIN-1 and restricts the early translocation of HSV-1 to the cell nucleus. **a**,**b**, Measurement of HSV-1–RFP intensity in HeLa cells overexpressing an EV, WT or various patient-specific *TMEFF1* mutants (**a**) or WT full-length *TMEFF1*, *TMEFF1* extracellular domain (EX) or transmembrane and intracellular domains (TM + IN) (**b**) at 10 h post-infection (hpi). Statistical analysis: Kruskal–Wallis tests with Dunn’s test for multiple comparisons; ***P < 0.001. a.u., arbitrary units. **c**,**d**, HEK293T cells were cotransfected with Flag-tagged NECTIN-1 and EV or WT *TMEFF1* plasmids (**c**) or with WT *TMEFF1* and EV or Flag-tagged NECTIN-1 plasmids (**d**), and subjected to immunoprecipitation (IP) with anti-TMEFF1 antibodies or anti-Flag antibody-conjugated agarose beads, and immunoblotting with anti-Flag or anti-TMEFF1 antibodies. **e**, HEK293T cells were infected with HSV-1 (MOI 1) and subjected to immunoprecipitation with mouse IgG isotype control or anti-NECTIN-1 antibody and immunoblotting with anti-NECTIN-1 or anti-TMEFF1 antibody. **f**, HEK293T cells were cotransfected with C-terminal Myc-tagged WT full-length or truncated (EX, TM + IN) TMEFF1 or N-terminal Myc-tagged WT full-length TMEFF1 plasmids with EV or Flag-tagged NECTIN-1 plasmids, and subjected to immunoprecipitation with anti-Myc antibody-conjugated agarose beads and immunoblotting with anti-Myc or anti-Flag antibody. **g**, HEK293T cells were cotransfected with the N-terminal Flag–GFP-tagged full-length or truncated (EX, TM + IN) NECTIN-1 plasmids with N-terminal Myc-tagged *TMEFF1* plasmids, and subjected to immunoprecipitation with anti-Flag antibody-conjugated agarose beads and immunoblotting with anti-Myc or anti-Flag antibody. **h**, HEK293T cells were cotransfected with the N-terminal Flag-tagged WT full-length or EX NECTIN-1 plasmids and N-terminal Myc-tagged WT full-length or EX TMEFF1 plasmids, and subjected to immunoprecipitation with anti-Flag antibody-conjugated agarose beads and immunoblotting with anti-Myc or anti-Flag antibody. **i**, HEK293T cells were cotransfected with the N-terminal Flag-tagged NECTIN-1 and EV, WT or various patient-specific mutant *TMEFF1* plasmids, and subjected to immunoprecipitation with anti-TMEFF1 antibody and immunoblotting with anti-TMEFF1 or anti-Flag antibody. The data shown in **a**–**i** are representative of three independent experiments. Source Data

**Extended Data Fig. 5:**
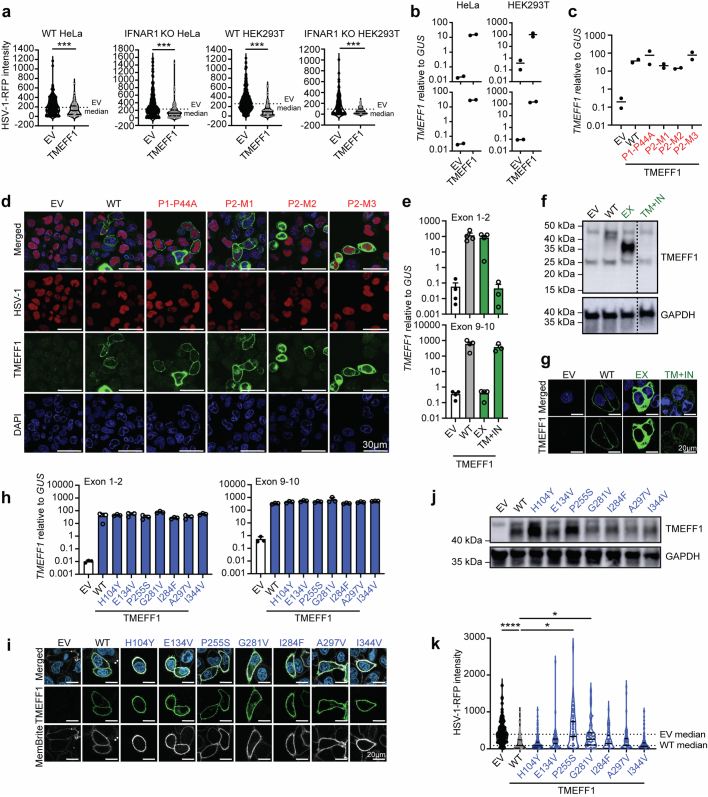
TMEFF1 restricts the early translocation of HSV-1 to the cell nucleus. **a**, RFP intensity in HeLa and HEK293T cells, as measured in an HSV-1-RFP nuclear translocation reporter assay 10 h after infection with an RFP-reporter HSV-1, on parental WT cells or IFNAR1 KO cells transfected with an empty vector (EV) or WT *TMEFF1* expression construct. HeLa or HEK293T cells were labeled with an anti-TMEFF1 antibody. RFP intensity was assessed under a confocal microscope. Statistical analysis was performed with two-tailed Mann-Whitney *U* tests. ***p-value < 0.001. The data shown are representative of three independent experiments. **b**, *TMEFF1* mRNA levels in HeLa (left panel) and HEK293T cells (right panel), either parental WT (top) or IFNAR1 KO (bottom), transfected with an EV or a WT *TMEFF1* expression plasmid, as measured by RT-qPCR. A probe targeting exons 1-2 of *TMEFF1* was used. **c**, *TMEFF1* mRNA levels in HeLa cells transfected with an EV, or a WT or mutant *TMEFF1* expression plasmid, as measured by RT-qPCR. A probe targeting exons 1-2 of *TMEFF1* was used. **d**, Representative images of HeLa cells transfected with an EV, or a WT or mutant *TMEFF1* expression plasmid, in an HSV-1-RFP nuclear translocation reporter assay. The cells were fixed and identified by DAPI staining (blue). HSV-1-RFP infection results in the expression of RFP, the levels of which were assessed in the nucleus at 10 hpi. **e**, *TMEFF1* mRNA levels in HeLa cells transfected with an EV, or a WT full-length (FL) *TMEFF1* or different domains of *TMEFF1* in an expression plasmid (EX: extracellular domain; TM + IN: transmembrane and intracellular domains), as measured by RT-qPCR. Two probes, targeting exons 1-2 (upper panel) and exons 9-10 (lower panel) of *TMEFF1*, were used. The data shown are representative of three independent experiments. **f**, TMEFF1 protein levels, as assessed by western blotting in HeLa cells transfected with an EV, or a WT FL or different domains of *TMEFF1* in an expression plasmid. **g**, TMEFF1 immunostaining in HeLa cells transfected with an EV, or a WT FL or different domains of *TMEFF1* in an expression plasmid. HeLa cells were labeled with anti-TMEFF1 antibody (green) and DAPI (blue) and *TMEFF1* overexpression was assessed under a confocal microscope. **h**, *TMEFF1* mRNA levels in HeLa cells transfected with an EV or with plasmids containing WT or gnomAD homozygous mutant (H104Y, E134V, P255S, G281V, I284F, A297V, I344V) *TMEFF1* cDNAs, as measured by RT-qPCR. Two probes, targeting exons 1-2 (upper panel) and exons 9-10 (lower panel) of *TMEFF1*, were used. **i**, TMEFF1 immunostaining in HeLa cells transfected with an EV or with plasmids containing WT or gnomAD homozygous mutant (H104Y, E134V, P255S, G281V, I284F, A297V, I344V) *TMEFF1* cDNAs. HeLa cells were labeled with anti-TMEFF1 antibody (green), membrane stain (MemBrite, white), and DAPI (blue) and *TMEFF1* overexpression was assessed under a confocal microscope. The data shown in **c**-**i** are representative of three independent experiments. **j**, TMEFF1 protein levels, as assessed by western blotting on HeLa cells transfected with an EV or with plasmids containing WT or gnomAD homozygous mutant (H104Y, E134V, P255S, G281V, I284F, A297V, I344V) *TMEFF1* cDNAs. **k**, Measurement of RFP intensity in an HSV-1-RFP nuclear translocation reporter assay, 10 h after infection, in HeLa cells cells transfected with an EV or with plasmids containing WT or gnomAD homozygous mutant (H104Y, E134V, P255S, G281V, I284F, I344V) *TMEFF1* cDNAs. HeLa cells were labeled with anti-TMEFF1 antibody and DAPI. RFP intensity was assessed under a confocal microscope. Statistical analysis was conducted with Kruskal-Wallis tests with Dunn’s test for multiple comparisons. ns: not significant, *p-value < 0.05; **** *p*-value < 0.0001. The data shown in **j**-**k** are representative of three independent experiments. Source Data

We then hypothesized that TMEFF1 might control HSV-1 infection through previously undescribed mechanisms, perhaps, given its surface expression, by serving as an HSV-1 restriction factor that blocks or impairs HSV-1 entry into cortical neurons. We first used *IFNAR1*-KO or parental HeLa and HEK293T cells, all of which are susceptible to HSV-1 infection, to investigate the effect of plasmid-mediated *TMEFF1* overexpression on HSV-1 infection. Interestingly, the transient overexpression of WT *TMEFF1* substantially decreased HSV-1 translocation to the nucleus in both cell types, with or without *IFNAR1* knockout, as shown by the lower density of HSV-1–RFP (red fluorescent protein) in the nuclei of cells with detectable TMEFF1 overexpression at the cell membrane 10 h after infection (Extended Data Fig. 5a,b). This indicates that WT *TMEFF1* overexpression blocked HSV-1 infection in all cells, regardless of their IFNAR1 status (with or without *IFNAR1* KO). We then assessed the density of HSV-1–RFP in the nuclei of HeLa cells with detectable WT or MT *TMEFF1* overexpression on the cell membrane or in the cytoplasm. Both P44A and P2-M3 completely lost the capacity to block HSV-1 infection (Fig. 4a and Extended Data Fig. 5c,d), whereas residual function was maintained for P2-M1 and P2-M2, indicating that, when expressed at high levels, the N-terminal TMEFF1, with an incomplete or truncated C-terminal region, was functional. Consistently, overexpression of the N-terminal extracellular domain of WT TMEFF1, but not that of the C-terminal intracellular domain with the transmembrane domain, decreased HSV-1 translocation to the nucleus to levels similar to those for full-length WT TMEFF1 (Fig. 4b and Extended Data Fig. 5e–g). These data indicate that the extracellular domain of TMEFF1, when properly expressed on the cell surface, is critical for restricting the early translocation of HSV-1 into the cell nucleus. They also indicate that P2-M3 is deleterious because of the absence of part of the TMEFF1 extracellular domain and/or the absence of the mutant protein on the plasma membrane (Fig. 2b–f), whereas P2-M2 is deleterious as a result of low levels of the protein, P2-M1 is functional by overexpression, and P1-MT is deleterious as a result of mechanisms not affecting the expression of TMEFF1 on the cell membrane. The other seven TMEFF1 variants (H104Y, E134V, P255S, G281V, I284F, A297V and I344V) found in the homozygous state in 11 of 807,162 individuals from the gnomAD database displayed normal expression and function when tested in vitro, except for P255S and G281V (each carried by a single individual in the homozygous state in gnomAD), which, like P1’s P44A variant, failed to restrict the early translocation of HSV-1 into the cell nucleus (Extended Data Fig. 5h–k). This made it possible to estimate exclusive enrichment in biallelic experimentally deleterious TMEFF1 variants in the HSE cohort relative to the gnomAD database representative of the general population (*P* = 1.9 × 10^−6^).

## TMEFF1 interacts with NECTIN-1

**Extended Data Fig. 6:**
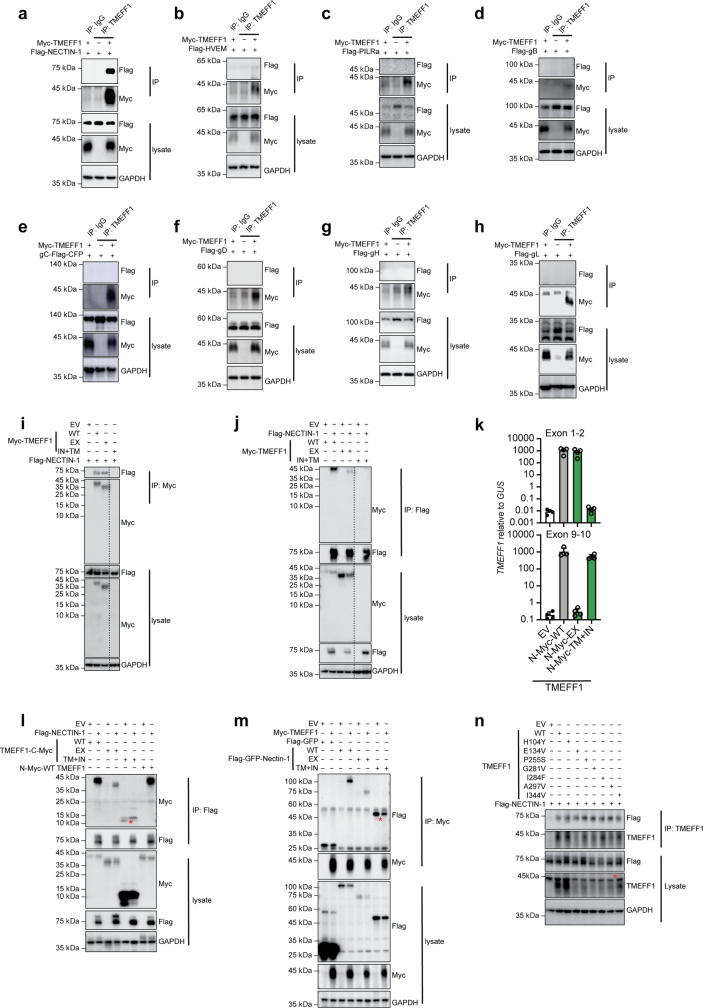
TMEFF1 does not interact with cellular receptors of HSV-1 other than nectin-1 or with HSV-1 glycoproteins. **a**-**h**, HEK293T cells were cotransfected with N-ter Myc-tagged TMEFF1 constructs and Flag-tagged NECTIN-1 (**a**), HVEM (**b**), PILRa (**c**), gB (**d**), gC (**e**), gD (**f**), gH (**g**), or gL (**h**). The cells were then subjected to immunoprecipitation (IP) with mouse IgG isotype control or anti-TMEFF1 Ab, and immunoblotting with anti-Flag or anti-Myc Abs. **i**-**j**, N-ter Flag-tagged NECTIN-1 constructs were co-expressed with N-ter Myc-tagged WT full-length (FL) or different domains (EX, TM + IN) of TMEFF1 in expression constructs in HEK293T cells, which were subjected to IP with anti-Myc Ab-conjugated agarose beads (**i**), or anti-Flag Ab-conjugated agarose beads (**j**), and immunoblotting with anti-Myc or anti-Flag Ab. **k**, *TMEFF1* mRNA levels, as determined by RT-qPCR, in HEK293T cells 24 h after transfection with N-ter Myc-tagged WT full-length (FL) or different domains (EX, TM + IN) of TMEFF1 in expression constructs. **l**, HEK293T cells were cotransfected with C-ter Myc-tagged WT FL, different domains (EX, TM + IN) of TMEFF1 in expression constructs, or an N-ter Myc-tagged WT FL TMEFF1 plasmid, together with EV or N-ter Flag-tagged NECTIN-1-expressing constructs. The cells were then subjected to IP with anti-Flag Ab-conjugated agarose beads and immunoblotting with anti-Myc or anti-Flag Ab. **m**, HEK293T cells were cotransfected with the N-ter Flag-GFP-tagged WT FL or different domains (EX, TM + IN) of NECTIN-1 in expression constructs together with N-ter Myc-tagged TMEFF1 constructs, and subjected to IP with anti-Myc Ab-conjugated agarose beads and immunoblotting with anti-Myc or anti-Flag Ab. For **l**-**m**, the red asterisk indicates non-specific binding. **n**, HEK293T cells were cotransfected with N-ter Flag-tagged NECTIN-1-expressing constructs and TMEFF1-expressing constructs containing the cDNA for the WT or homozygous TMEFF1 variants from the gnomAD database (H104Y, E134V, P255S, G281V, I284F, A297V, I344V). The cells were then subjected to IP with anti-Flag Ab-conjugated agarose beads, and immunoblotting with anti-TMEFF1 or anti-Flag antibodies. The red asterisk indicates non-specific binding. The data shown in **a**-**n** are representative of three independent experiments. Source Data

We assessed HSV-1 translocation to the cell nucleus 10 h after infection, a time point corresponding to a single cycle of virus production in the nucleus. Enhanced translocation of the virus to the nucleus may therefore be considered indicative of enhanced virus entry through the cell membrane followed by translocation to the nucleus. Several HSV-1 and cellular proteins have been shown to be essential for the entry of HSV-1 into human cells, including neurons, through their mediation of virus binding and virus–cell fusion^40^. We tested the hypothesis that TMEFF1 interacts with one or more of these viral or cellular proteins, thereby interfering with HSV-1 entry into neurons, by performing co-immunoprecipitation experiments based on the plasmid transfection-mediated overexpression of TMEFF1 and any of the five key HSV-1 glycoproteins (gB, gC, gD, gH and gL) and the three well-characterized neuronal HSV-1 receptors (NECTIN-1, HVEM and PILRa) in HEK293T cells. Remarkably, TMEFF1 was co-immunoprecipitated with NECTIN-1 (Fig. 4c,d and Extended Data Fig. 6a), but not with HVEM, PILRa or the five viral glycoproteins (Extended Data Fig. 6b–h), indicating a direct or indirect interaction of TMEFF1 with NECTIN-1. Cell-endogenous TMEFF1 was also co-immunoprecipitated with cell-endogenous NECTIN-1 in HEK293T cells, and HSV-1 infection did not disrupt this interaction (Fig. 4e). The TMEFF1 extracellular domain was detected with untagged constructs or with constructs tagged at the N terminus or C terminus, whereas the TMEFF1 transmembrane domain and its intracellular tail were detected only when a C terminus-tagged construct was used (Fig. 4f–h and Extended Data Fig. 6i–m). We further showed that the extracellular domain interacted with full-length NECTIN-1 or with the extracellular domain of NECTIN-1 (which is identical in the three natural isoforms of NECTIN-1) but not with the transmembrane domain or intracellular tail of NECTIN-1, and that no such interactions were observed with the transmembrane domain or intracellular tail of TMEFF1 (Fig. 4f–h and Extended Data Fig. 6i–m). Finally, the mutant TMEFF1 proteins of P1 and P2 were co-immunoprecipitated normally (P44A and P2-M1), weakly (P2-M2) or not at all (P2-M3) with NECTIN-1 (Fig. 4i), indicating that the molecular mechanisms that impair the inhibition of HSV-1 infection by these mutant TMEFF1 proteins differ quantitatively (P2-M2) and qualitatively (P1 MT and P2-M3). We also tested the other seven variants (H104Y, E134V, P255S, G281V, I284F, A297V and I344V) present in the homozygous state in gnomAD, all of which were normally co-immunoprecipitated with NECTIN-1 (Extended Data Fig. 6n). These findings indicate that the extracellular domain of TMEFF1 interacts with the extracellular domain of the HSV-1 receptor NECTIN-1, and that this interaction may interfere with the entry of HSV-1 into cells using NECTIN-1.

## TMEFF1 impairs HSV-1 entry to neurons

**Fig. 5:**
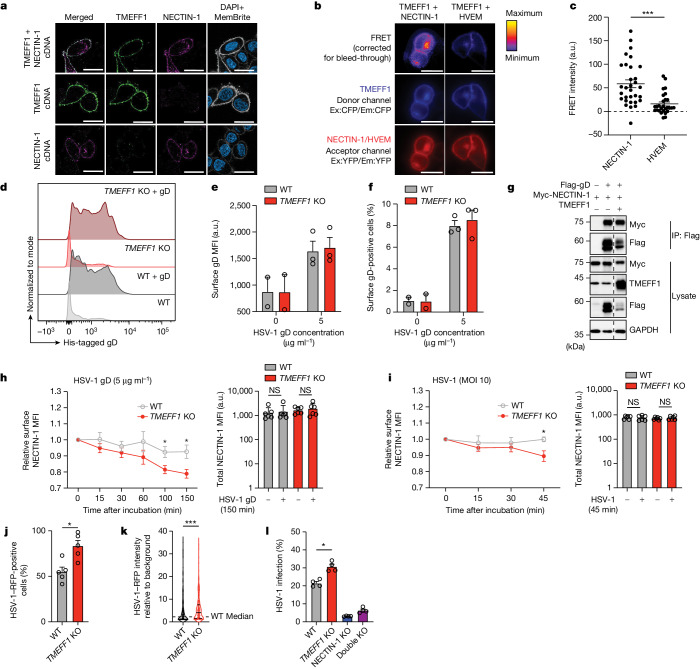
TMEFF1 interaction with NECTIN-1 on the cell surface impairs HSV-1 entry. **a**, TMEFF1 and NECTIN-1 localization in HeLa cells after cotransfection or single transfection with the TMEFF1 and NECTIN-1 plasmids. Green, TMEFF1; purple, NECTIN-1; blue, DAPI; white, MemBrite. Scale bars, 20 μm. **b**, HeLa cells were cotransfected with CFP-tagged TMEFF1 and YFP-tagged NECTIN-1 or HVEM, and subjected to FRET imaging. Scale bars, 20 μm. CFP, cyan fluorescent protein; YFP, yellow fluorescent protein; Ex, excitation; Em, emission. **c**, Bleed-through-corrected FRET at the cell surface was quantified. **d**–**f**, Histograms of the surface His-tagged gD signal (**d**), the MFI of surface gD binding (**e**) and the percentage of surface gD-positive cells (**f**) after incubation with a His-tagged gD for 150 min in WT or *TMEFF1*-KO HEK293T cells stably expressing NECTIN-1. **g**, HEK293T cells were cotransfected with Flag-tagged gD, Myc-tagged NECTIN-1 and EV or TMEFF1 plasmids, then subjected to immunoprecipitation with anti-Flag antibody-conjugated agarose beads, and immunoblotting with anti-Flag, anti-Myc and anti-TMEFF1 antibodies. The data shown in **a**–**g** are representative of three independent experiments. **h,i**, MFI of surface NECTIN-1 after gD treatment (**h**) or HSV-1 infection (**i**) relative to untreated cells (left) and MFI of total NECTIN-1 in the presence or absence of gD treatment or HSV-1 infection (right) on WT or *TMEFF1*-KO HEK293T cells. **j**, HSV-1–RFP infection rates in WT and *TMEFF1*-KO HEK293T cells 8 hours after infection. **k**, HSV-1–RFP intensity in WT and *TMEFF1*-KO HEK293T cell nucleus 8 hours after infection. Data are shown as median ± interquartile range and are representative of five independent experiments. **l**, HSV-1–RFP infection rates in WT, *TMEFF1* KO, *NECTIN-1* KO and *TMEFF1*-and-*NECTIN-1* double-KO HEK293T cells, as assessed by flow cytometry 8 hours after infection. Data are shown as mean ± s.e.m. from three (**b**, **c**, **e** and **f**), six (**h** and **i**), five (**j**) or four (**l**) independent experiments. Statistical analysis was done for **c**, **h**, **i**, **j**, **k** and **l** using two-tailed Mann–Whitney *U* tests; *P < 0.05; ***P < 0.001. Source Data

**Extended Data Fig. 7:**
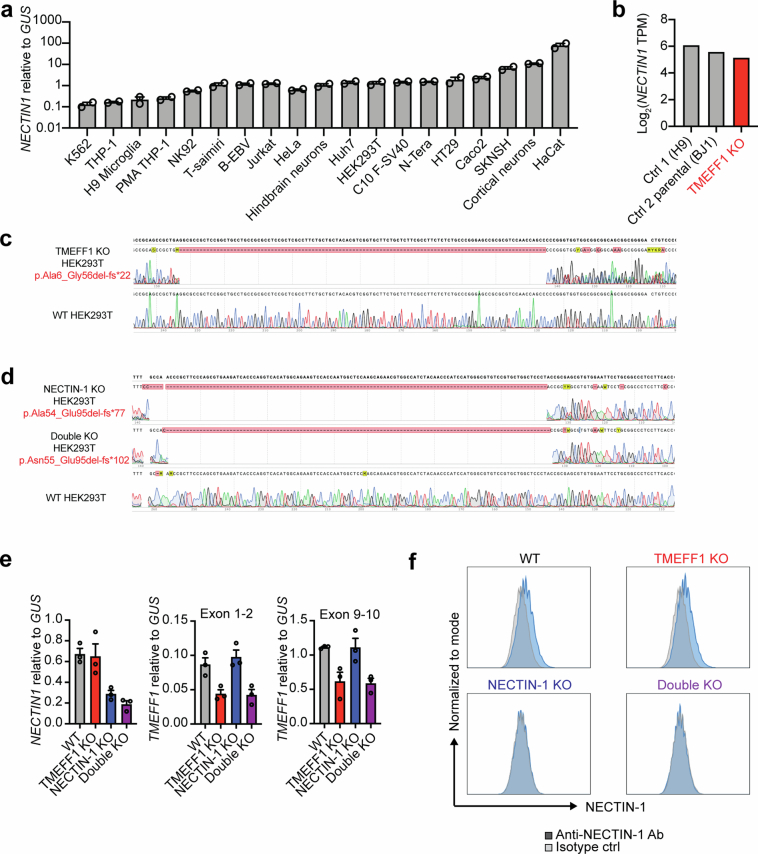
Characterization of gene-edited HEK293T lacking TMEFF1 or NECTIN-1. **a**, Levels of *NECTIN1* mRNA, as determined by RT-qPCR, in various human cell lines or primary cells. The data shown are from two independent experiments. **b**, Abundance of the canonical *NECTIN1* transcript (isoform 1, delta, ENST00000264025.8) as assessed by RNAseq, in cortical neurons derived from gene-edited line of TMEFF1 KO hPSCs, and from healthy controls (H9, BJ1). *NECTIN1* transcripts for isoform 2 (ENST00000341398.6) and 3 (ENST00000340882.2) were undetectable. **c**, Electropherogram showing the *TMEFF1* gDNA sequence at the sgRNA target region in WT and TMEFF1 KO HEK293T cells. **d**, Electropherogram showing the *NECTIN1* gDNA sequence at the sgRNA target region in WT, NECTIN-1 KO, and TMEFF1 and NECTIN-1 double KO HEK293T cells. **e**, Levels of *NECTIN1* (left panel) and *TMEFF1* (center and right panels) mRNA, as determined by RT-qPCR, in WT, TMEFF1 KO, NECTIN-1 KO, and TMEFF1 and NECTIN-1 double KO HEK293T cells. Two probes, targeting exons 1-2 (center panel) and exons 9-10 (right panel), were used for *TMEFF1*. **f**, Cell-surface NECTIN-1 protein expression assessed by flow cytometry in WT, TMEFF1 KO, NECTIN-1 KO, and TMEFF1 and NECTIN-1 double KO HEK293T cells. Source Data

**Extended Data Fig. 8:**
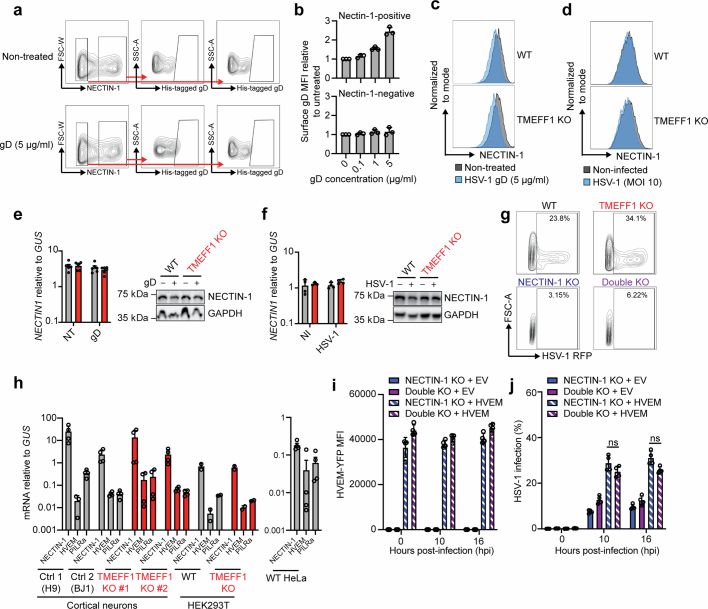
TMEFF1 restricts the early translocation of HSV-1 to the cell nucleus by interfering with gD-NECTIN-1-mediated viral entry. **a**, Representative gating strategy for HEK293T cells stably expressing NECTIN-1, not treated or treated with recombinant His-tagged HSV-1 gD for 150 min. **b**, Mean fluorescence intensity (MFI) of surface gD binding in NECTIN-1-positive or NECTIN-1-negative fractions of HEK293T cells stably expressing NECTIN-1, following incubation with different concentrations of His-tagged gD (gD-His-Tag) for 150 min. **c**, Histogram of surface NECTIN-1 expression in WT and TMEFF1 KO HEK293T cells incubated with recombinant His-tagged HSV-1 gD (5 µg/ml) for 150 min. The data shown are representative of three independent experiments. **d**, Histogram of surface NECTIN-1 expression in WT and TMEFF1 KO HEK293T cells infected with HSV-1 (MOI 10) for 45 min. The data shown are representative of three independent experiments. **e**, *NECTIN1* mRNA levels (left panel) as determined by RT-qPCR in total RNA, and protein expression in cell total lysates as assessed by immunoblotting (right panel), in TMEFF1 KO or parental WT HEK293T cells upon gD treatment for 150 min or without treatment. **f**, *NECTIN1* mRNA levels (left panel) as determined by RT-qPCR in total RNA, and protein expression in cell total lysates as assessed by immunoblotting (right panel), in TMEFF1 KO or parental WT HEK293T cells after infection with HSV-1 for 45 min, or without infection. **g**, Representative contour plots of the RFP signal in WT, TMEFF1 KO, NECTIN-1 KO, and TMEFF1 and NECTIN-1 double KO HEK293T cells infected with HSV-1-RFP (MOI 10) at 8 hpi. **h**, Basal mRNA levels for *NECTIN-1*, *HVEM*, and *PILRa*, as assessed by RT-qPCR, in WT parental or TMEFF1 KO hPSC-derived cortical neurons and HEK293T cells (left panel), and WT HeLa cells (right panel). The data shown are the mean ± SEM from three independent experiments. **i**, MFI of N-ter YFP-tagged HVEM on the cell surface in NECTIN-1 KO and TMEFF1 and NECTIN-1 double KO HEK293T cells transfected with an empty vector (EV), or N-ter YFP-tagged HVEM-expressing plasmid. The data shown are the mean ± SEM from four independent experiments. **j**, Percentage of HSV-1-positive cells, as assessed in an assay of HSV-1 translocation to the cell nucleus 10 h after infection with an RFP-reporter HSV-1, in NECTIN-1 KO or TMEFF1 and NECTIN-1 double KO HEK293T cells transfected with an empty vector (EV) or HVEM-expressing plasmid. The data are presented as the mean ± SEM from four independent experiments. Statistical analysis was conducted with Kruskal-Wallis tests with Dunn’s test for multiple comparisons. ns: not significant. Source Data

As with TMEFF1, NECTIN-1 is expressed on the cell surface^41^ and is strongly expressed on neurons (Extended Data Fig. 7a,b). When overexpressed in transfected HeLa cells, TMEFF1 and NECTIN-1 colocalized on the cell membrane, and co-expression with TMEFF1 did not affect the cell-surface expression pattern of NECTIN-1 (Fig. 5a). Using a Förster resonance energy transfer (FRET) assay, we then showed that WT TMEFF1, when overexpressed, interacts closely with NECTIN-1, but not with HVEM, at the cell surface (Fig. 5b,c). Consistently, cell-endogenous expression of NECTIN-1 at the surface of HEK293T cells was not altered by *TMEFF1* KO (Extended Data Fig. 7c–f). HSV-1 gD can bind to its cellular receptors, including NECTIN-1 and HVEM, to initiate membrane fusion and virus entry^41–43^. We found that stably expressed NECTIN-1 bound to recombinant HSV-1 gD at the cell surface, and that levels of gD binding were similar in *TMEFF1* KO and parental WT cells (Fig. 5d–f and Extended Data Fig. 8a,b), indicating that TMEFF1 does not compete with HSV-1 gD for binding to NECTIN-1 at the cell surface. This result was confirmed by the co-immunoprecipitation of gD with NECTIN-1 in the presence of TMEFF1 (Fig. 5g). It has been shown that recombinant gD or HSV-1 infection results in the gD-dependent internalization of NECTIN-1, indicating virus–cell membrane fusion and HSV-1 entry^44–46^. As expected, gD treatment or HSV-1 infection of HEK293T cells rapidly decreased the cell-surface expression of NECTIN-1 (Fig. 5h,i and Extended Data Fig. 8c–f). Moreover, this decrease in NECTIN-1 levels at the cell surface was even more marked in *TMEFF1*-KO cells (Fig. 5h,i and Extended Data Fig. 8c–f), indicating that the interaction between TMEFF1 and NECTIN-1 at the cell surface interfered with virus–cell membrane fusion following gD–NECTIN-1 binding, thereby impairing HSV-1 entry into the cells. Indeed, *TMEFF1* KO rendered HEK293T cells susceptible to the early translocation of HSV-1 to the nucleus (Fig. 5j–l), and *NECTIN-1* KO in *TMEFF1*-KO or parental cells rendered both cell types equally resistant to HSV-1 infection (Fig. 5l and Extended Data Figs. 7c–f and 8g). HVEM is another HSV-1 receptor that can bind gD^42,43^ and is expressed at low levels in cortical neurons, HEK293T and HeLa cells (Extended Data Fig. 8h). Its overexpression rendered *NECTIN-1*-KO and *NECTIN-1*–*TMEFF1* double-KO cells slightly more susceptible to HSV-1 (Extended Data Fig. 8i,j), confirming that TMEFF1 specifically impairs NECTIN-1-mediated, but not HVEM-mediated, HSV-1 entry. Taken together, these data indicate that the extracellular domain of TMEFF1 interacts with the extracellular domain of the HSV-1 receptor NECTIN-1 on the cell surface, thereby interfering with the HSV-1 gD–NECTIN-1-mediated fusion of the virus with the cell, restricting the entry of HSV-1 into neurons.

## HSV-1 entry in TMEFF1-deficient neurons

**Fig. 6:**
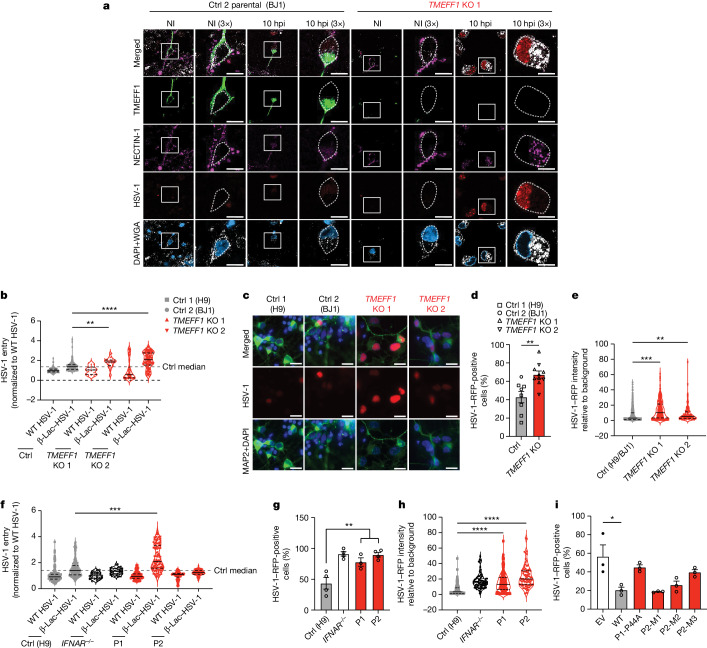
Enhanced HSV-1 entry results in greater viral susceptibility in TMEFF1-deficient cortical neurons. **a**, Representative images of healthy control (Ctrl 2 parental-BJ1) and *TMEFF1*-KO hPSC-derived cortical neurons, stained for endogenous TMEFF1 (green), NECTIN-1 (purple), chromosomes (DAPI, blue) and cell membrane (WGA, white) before and 10 h after infection (hpi) with an RFP reporter HSV-1 (red). The dashed grey line is located immediately beneath the WGA-stained cell membrane. The areas in white squares are enlarged in the image on the right. Scale bar, 10 μm. The images are representative of three independent experiments. NI, non-infected. **b**, Comparison of HSV-1 entry into heathy control (Ctrl 1-H9, Ctrl 2 parental-BJ1) and *TMEFF1*-KO hPSC-derived cortical neurons in a β-lactamase assay (449/520 nm). Data are shown as median ± interquartile range and are representative of three independent experiments. **c**, Representative images of hPSC-derived cortical neurons in an HSV-1–RFP cell nuclear translocation reporter assay 10 h after infection. Neurons were identified by staining for microtubule-associated protein 2 (MAP2, green) and chromosomes (DAPI, blue). Scale bar, 10 μm. **d**,**e**, Percentage of HSV-1-positive (**d**) and cell nuclear RFP intensity (**e**) of healthy control and *TMEFF1*-KO hPSC-derived cortical neurons 10 h after infection. **f**, Comparison of HSV-1 entry into healthy control (H9), *IFNAR1*^−/−^, P1 and P2 hPSC-derived cortical neurons in the β-lactamase assay. **g**,**h**, Percentage of HSV-1-positive (**g**) and cell nuclear RFP intensity (**h**) of healthy control, *IFNAR1*^−/−^, P1 and P2 hPSC-derived cortical neurons 10 h after infection. **i**, Percentage of HSV-1-positive *TMEFF1*-KO cortical neurons transduced with EV, WT *TMEFF1* or patient-specific *TMEFF1* variant cDNA 10 h after infection. Data are shown as mean ± s.e.m. (**d**, **g** and **i**) or median ± interquartile range (**e**, **f** and **h**) from four (**d** and **e**) or three (**f**, **g**, **h** and **i**) independent experiments. Statistical analysis was done for **d**, **g** and **i** using two-tailed Mann–Whitney *U* tests, and for **b**, **e**, **f** and **h** using Kruskal–Wallis tests with Dunn’s test for multiple comparisons. *P < 0.05; **P < 0.01; ***P < 0.001; ****P < 0.0001. Source Data

**Extended Data Fig. 9:**
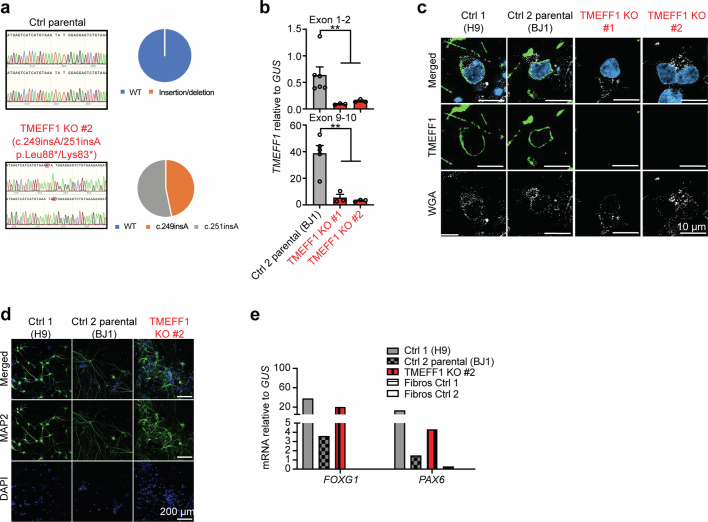
Characterization of additional gene-edited line of hPSC-derived cortical neurons lacking TMEFF1. **a**, Electropherogram representation (left panels) of the CRISPR-Cas9-introduced compound-heterozygous *TMEFF1* mutations confirmed by Sanger sequencing on genomic DNA from an additional gene-edited line of TMEFF1 KO hPSCs (TMEFF1 KO #2). Sequencing results for the parental line (Ctrl parental, BJ1) are also shown. The relative abundance of WT and mutated *TMEFF1* cDNAs generated from mRNA extracted from the parental control and TMEFF1 KO hPSC clone #2 was assessed by TOPO-TA cloning and is shown in the panels on the right. **b**, *TMEFF1* mRNA levels were determined by RT-qPCR on cortical neurons from control parental clones and two different clones of TMEFF1 KO human pluripotent stem cells (hPSCs). Two probes, targeting exons 1-2 (upper panel) and exons 9-10 (lower panel) of *TMEFF1*, were used. The data shown are the mean ± SEM from three independent experiments. Statistical analysis was conducted with two-tailed Mann-Whitney *U* tests. **p-value < 0.01. **c**, TMEFF1 protein expression was studied by confocal microscopy on cortical neurons derived from healthy control H9, control parental (BJ1) and TMEFF1 KO hPSCs. Cells were fixed and stained for TMEFF1 with anti-TMEFF1 antibody (green), the cell membrane was stained with WGA (white), and chromosomes were stained with DAPI (blue). **d**, Representative images of cortical neurons from controls (Ctrl 1-H9, Ctrl 2 parental-BJ1) and TMEFF1 KO hPSC line #2. Cells were fixed and stained with DAPI (blue) and with microtubule-associated protein 2 (MAP2, green) as a neuron-specific marker. **e**, *FOXG1* and *PAX6* mRNA levels, as measured by RT-qPCR, in cortical neurons from a control and TMEFF1 KO hPSC line #2. SV40-transformed fibroblasts from healthy controls (Fibros ctrl 1, Fibros ctrl 2) were used as a negative control in this assay. Source Data

**Extended Data Fig. 10:**
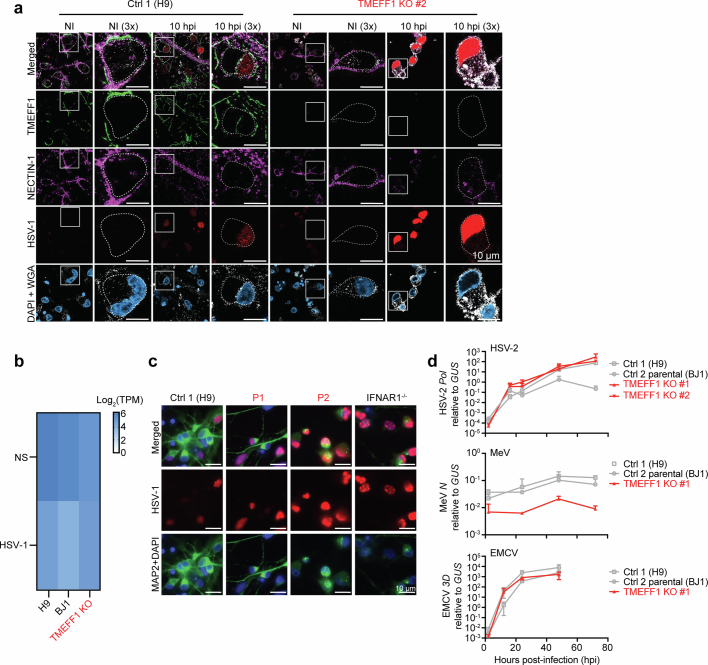
Enhanced HSV-1 entry resulting in greater viral susceptibility in cortical neurons lacking TMEFF1. **a**, Immunostaining of hPSC-derived cortical neurons from healthy control (H9) and TMEFF1 KO hPSCs (TMEFF1 KO #2) for endogenous TMEFF1 (green), NECTIN-1 (purple), AT-rich DNA (DAPI, blue) and the cell membrane (WGA, white) before and 10 h after infection with an RFP-reporter HSV-1 (red). The dashed white line is immediately beneath the WGA-stained cell membrane. **b**, Abundance of *NECTIN1* mRNA, as assessed by RNAseq, in WT controls (Ctrl1-H9, Ctrl2 parental-BJ1) and TMEFF1 KO hPSC-derived cortical neurons infected with HSV-1 for 24 h or left unstimulated (NS). **c**, Immunostaining of hPSC-derived cortical neurons from a healthy control (Ctrl1-H9), an IFNAR1^−/−^ patient, and P1 and P2 with *TMEFF1* mutations, in a reporter assay for the nuclear translocation of HSV-1, 10 h after infection with an RFP-reporter HSV-1. Neurons were fixed and identified by staining for a neuron-specific microtubule-associated protein 2 (MAP2, green) and with DAPI (blue). HSV-1-RFP infection results in the expression of RFP, which was detected in the nucleus 10 hpi. **d**, Replication levels for HSV-2, measles virus (MeV), or EMCV, following infection at various time points as indicated, in hPSC-derived cortical neurons from healthy controls (Ctrl1-H9, Ctrl2 parental-BJ1), or TMEFF1 KO hPSCs. RT-qPCR was performed with the SYBR green assay, to assess HSV-2 polymerase (*Pol*), MeV nucleocapsid (*N*) or EMCV *3D* gene expression indicative of viral replication levels. The data are presented as the mean ± SD and are representative of four independent experiments. Source Data

**Extended Data Fig. 11:**
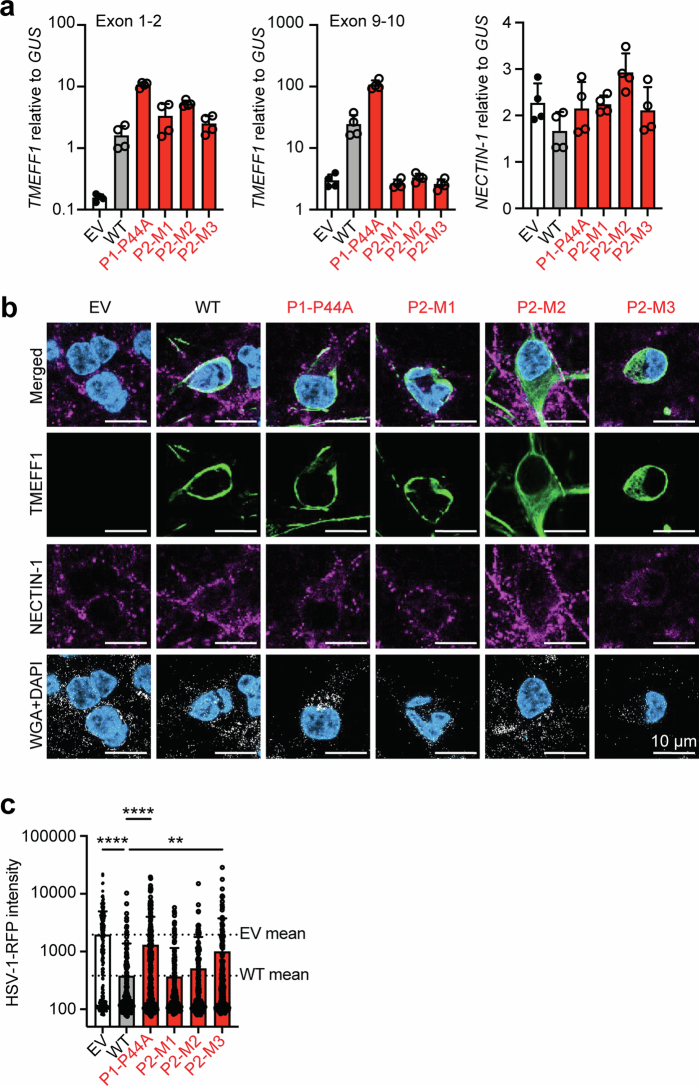
Exogenous expression of wild-type but not mutant TMEFF1 renders TMEFF1 KO neurons normally resistant to HSV-1 infection. **a**, *TMEFF1* and *NECTIN1* mRNA levels, as measured by RT-qPCR, in TMEFF1 KO cortical neurons transduced with an EV, WT TMEFF1 or patient-specific mutant TMEFF1-expressing lentivirus. Two probes, targeting exons 1-2 (left) and exons 9-10 (center) of *TMEFF1*, were used. The data shown are representative of two independent experiments. **b**, Immunostaining of exogenous TMEFF1 (green), endogenous NECTIN-1 (purple), AT-rich DNA (DAPI, blue) and the cell membrane (WGA, white), for hPSC-derived TMEFF1 KO cortical neurons transduced with EV, WT TMEFF1 or patient-specific mutant TMEFF1-expressing lentiviruses. The data shown are representative of three independent experiments. **c**, Measurement of RFP intensity, 10 h after infection with HSV-1-RFP, in TMEFF1 KO cortical neurons transduced with EV, WT TMEFF1 or patient-specific mutant TMEFF1-expressing lentiviruses. Cortical neurons were labeled with anti-TMEFF1 antibody (green) and DAPI (blue). RFP intensity was assessed under a confocal microscope. HSV-1 infection results in the expression of RFP, which is detected in the nucleus at 10 hpi. Statistical analysis was conducted with Kruskal-Wallis tests with Dunn’s test for multiple comparisons. **p-value < 0.01; ****p-value < 0.0001. The data are presented as the mean ± SEM from three independent experiments. Source Data

We then studied the HSV-1 infection cycle, from virus entry to nuclear translocation, in cortical neurons derived from *TMEFF1*-KO and parental WT hPSCs (Fig. 6a–e and Extended Data Figs. 3a–c, 9a–e and 10a) and from hPSCs from the two patients and healthy controls (Fig. 6f–h and Extended Data Fig. 3d,e). First, as in HeLa cells following overexpression (Fig. 5a), cell-endogenous TMEFF1 and NECTIN-1 colocalized on the neuron cell surface (Fig. 6a and Extended Data Fig. 10a). Similar levels of *NECTIN1* mRNA were detected in *TMEFF1*-KO and parental WT hPSC-derived cortical neurons in the presence and absence of HSV-1 infection (Extended Data Fig. 10b). HSV-1 infection decreased the cell-surface expression of NECTIN-1, this decrease being more pronounced in *TMEFF1*-KO neurons (Fig. 6a and Extended Data Fig. 10a), consistent with the data for cell-surface NECTIN-1 expression in HEK293T cells (Fig. 5i and Extended Data Fig. 8d). In the absence of an appropriate method for quantifying cell-surface NECTIN-1 expression in neurons, we investigated whether a lack of TMEFF1 resulted in enhanced HSV-1–cell membrane fusion and virus entry during the first few hours of viral infection by assessing HSV-1 entry with a modified HSV-1 encoding a fusion of β-lactamase to the viral tegument protein pUL47, making it possible to quantify CCF2 cleavage following the entry of HSV-1 into the cells^47^. By 1 h after infection, higher levels of HSV-1 entry were observed in two clones of *TMEFF1*-KO neurons than in WT parental neurons or WT neurons differentiated from another healthy control hPSC line (Fig. 6b). Enhanced HSV-1 entry into *TMEFF1*-KO neurons was associated with higher rates of translocation of the virus to the nucleus 10 h after infection (Fig. 6c–e). Similarly, cortical neurons derived from the hPSCs of P1 also displayed enhanced HSV-1 entry relative to neurons from healthy controls 1 h after infection (Fig. 6f), followed by increased nuclear translocation 10 h after infection (Fig. 6g,h and Extended Data Fig. 10c). In P2 neurons, HSV-1 entry was not enhanced 1 h after infection, consistent with the finding that, as well as the loss-of-function P2-M3 TMEFF1, P2’s cells also expressed two neutral or hypomorphic mutant forms of TMEFF1: P2-M1, which after overexpression was present in normal amounts on the cell membrane and displayed normal levels of interaction with NECTIN-1, and P2-M2, which was less abundant on the cell membrane and also had lower levels of interaction with NECTIN-1 (Fig. 2d–f and Fig. 4i). However, the translocation of HSV-1 to the nucleus was clearly increased 10 h after infection in P2’s neurons (Fig. 6g,h and Extended Data Fig. 10c), indicating that HSV-1 entry may have been enhanced in P2’s neurons, but at a time point later than 1 h after infection. By contrast, *TMEFF1*-KO neurons displayed similar levels (for EMCV and the measles virus) or slightly higher (HSV-2) levels of infection with the other viruses tested than did WT parental cells (Extended Data Fig. 10d). This finding is consistent with the different (EMCV and measles virus) or partly overlapping (HSV-2, which also uses NECTIN-1) routes of entry of these viruses relative to HSV-1 (ref. ^43^). Finally, the overexpression of WT *TMEFF1*, but not of P1 MT or P2-M3 *TMEFF1*, rescued the phenotype of early translocation to the cell nucleus of HSV-1 in *TMEFF1*-KO neurons (Fig. 6i and Extended Data Fig. 11a–c). These data are consistent with the restriction of HSV-1 entry into cells by WT but not by MT TMEFF1 in HeLa cells in vitro (Fig. 4a), and the enhanced HSV-1 replication phenotype observed in cortical neurons with TMEFF1 deficiency (Fig. 3d,g), confirming that TMEFF1 restricts HSV-1 entry by its expression on the surface of cortical neurons.

## Discussion

Autosomal recessive TMEFF1 deficiency is a new genetic aetiology of HSE. About 5% of children with HSE have experimentally proven autosomal recessive or autosomal dominant deficiency of the TLR3–IFNα/β circuit, which governs cell-intrinsic immunity in cortical neurons^8–17,24,25^, and another roughly 2% have autosomal dominant or autosomal recessive deficiencies that govern other antiviral mechanisms in the forebrain (snoRNA31, RIPK3 and TFIIIA) or brainstem (DBR1)^18,21–23^. As for other genetic aetiologies of HSE, the clinical penetrance of TMEFF1 deficiency seems to be incomplete, which is consistent with the sporadic nature of HSE^4^. The mechanisms of incomplete penetrance across genetic aetiologies are unknown, but viral inoculum and age at infection may be key contributors^2,39^. Contrary to the findings obtained for snoRNA31 and RIPK3 deficiencies to date, both TLR3–IFN and DBR1 deficiencies can underlie infections of the CNS with viruses other than HSV-1 (refs. ^23,48^). Unlike TLR3–IFN deficiency, which has also been reported in patients with severe viral diseases of tissues other than the brain, such as severe influenza and COVID-19 pneumonia^49,50^, deficiencies of snoRNA31, RIPK3 and DBR1 have not yet been detected in patients with viral infections of other organs. TFIIIA deficiency also impairs adaptive immunity, thereby underlying various other infections^18^. Inherited TMEFF1 deficiency is also unlikely to underlie viral diseases outside the CNS, given the preferential expression of TMEFF1 in the brain. Nevertheless, TMEFF1 deficiency could potentially underlie other viral infections of the CNS, because its cellular effect may not necessarily be specific for HSV-1. Other viruses, such as HSV-2 and varicella zoster virus, which also partly use NECTIN-1 to enter cells^40,51^, may cause encephalitis in TMEFF1-deficient patients. Future studies should search for *TMEFF1* variants in other patients suffering from HSE and other viral infections of the CNS.

We show that human *TMEFF1* encodes a type I IFN-independent, cortical neuron- and CNS-intrinsic restriction factor that is effective against HSV-1. TLR3 and type I interferons control a general antiviral mechanism in cortical neurons; RIPK3 controls HSV-1 infection in cortical neurons through cell death-mediated antiviral immunity, whereas the molecular mechanisms by which snoRNA31 and DBR1 control antiviral immunity in the human brain remain unclear. By contrast, TMEFF1 operates by impairing the entry of HSV-1 into cortical neurons, possibly accounting for the sanctuary status of the CNS, which is often protected from HSV-1, even in patients with disseminated HSV-1 as the result of profound leukocyte deficiencies^7^. Our findings also indicate that the extracellular domain of TMEFF1 interacts with the extracellular domain of NECTIN-1, a key cellular receptor for HSV-1 (ref. ^40^), at the cell surface. A deficiency of TMEFF1 does not seem to result in excessive binding of viral gD to the surface of cells expressing NECTIN-1. However, TMEFF1 deficiency enhances the gD–NECTIN-1 binding-mediated fusion of the virus with the cell, resulting in the entry of excessive numbers of viruses, thereby increasing HSV-1 translocation to the nucleus and replication (Supplementary Fig. 1). The molecular mechanisms by which fusion of the virus with the cell is impaired by TMEFF1 may involve disruption of the viral fusion complex, which contains gD, other viral glycoproteins, NECTIN-1 and other cellular proteins^42,43^. Alternative mechanisms for impaired HSV-1 internalization following gD and NECTIN-1 binding cannot be excluded^52^. More detailed mechanistic investigations are warranted.

We are aware of only one similar example of a human viral restriction factor that is essential in vivo, and that is the EVER–CIB1 complex, which protects keratinocytes from β-HPVs; inherited deficiencies of this complex underlie epidermodysplasia verruciformis^53^. Both TMEFF1 and the EVER–CIB1 complex are type I IFN-independent. These observations indicate that the surprisingly narrow range of severe viral diseases in patients with autosomal recessive IRF7, IFNAR1 or IFNAR2 deficiency^54–57^, which seem to be restricted to certain viral diseases of the lungs and brain, and adverse reactions to certain live-attenuated viruses, may be due to the existence of many other type I IFN-independent restriction factors specific for different viruses in different tissues. These two restriction factors mirror the human viral-susceptibility factors CCR5 and FUT2, which render CD4^+^ T cells and intestinal epithelial cells permissive to HIV and norovirus, respectively, and deficiencies of which protect against infection^58,59^. Finally, an intriguing implication of our study is that an absence of TMEFF1 or its weak expression in cells outside the CNS may contribute to the vulnerability of these cells to HSV-1. This, in turn, indicates that the extracellular domain of TMEFF1, if produced in a soluble form, would be of potential therapeutic value for combating infections with viruses such as HSV-1, and perhaps also HSV-2, in the CNS or other organs. As proof of principle, CCR5-blocking agents have already been widely developed for use in treatment strategies against HIV^60^. The discovery that human TMEFF1 is a cortical neuron-specific restriction factor for HSV-1 in vitro and in vivo has exciting biological and medical implications.

## Methods

### Human subjects

Informed consent was obtained in France in accordance with local regulations and a human-subjects research protocol approved by the institutional review board (IRB) of the Institut National de la Santé et de la Recherche Médicale (INSERM). Experiments were done in the United States and France in accordance with local regulations and with the approval of the IRB of The Rockefeller University and INSERM, respectively, to conduct human genetic and immunological studies, including those that use hPSC-derived tissue-specific cells for research purposes. Approval was obtained from the French Ethics Committee (Comité de Protection des Personnes), the French National Agency for Medicine and Health Product Safety, and INSERM in Paris (protocol C10-13) and the Rockefeller University Institutional Review Board in New York (protocol SHZ-0676). The hPSC-related work was approved by the Tri-Institutional Stem Cell Initiative Embryonic Stem Cell Research Oversight Committee (protocol 2021-05-003).

### Cell culture

Primary human fibroblasts were obtained from skin biopsy specimens from controls, P1 and P2, and were cultured in DMEM (GIBCO BRL, Invitrogen) supplemented with 10% fetal calf serum (FCS) (GIBCO BRL, Invitrogen). Immortalized SV40-transformed fibroblast cell lines were created by using 4 mg of a plasmid containing T-antigen DNA to transfect about 5 million cells by electroporation. The cells were then placed in two fresh 75 cm^2^ flasks each containing 12 ml DMEM (GIBCO BRL, Invitrogen) supplemented with 10% FCS (GIBCO BRL, Invitrogen). SV40 fibroblast clones appeared after about 15 days. These clones were cultured and passaged for experimental use. HEK293T cells (ATCC) and HeLa cells (ATCC) were maintained in DMEM supplemented with 10% FCS. All cells were regularly checked to ensure they were negative for mycoplasma.

### Whole-exome sequencing

Genomic DNA was isolated by phenol-chloroform extraction from peripheral blood cells or primary fibroblasts from the patient. DNA (3 µg) was sheared with a Covaris S2 Ultrasonicator (Covaris). An adapter-ligated library was prepared with the TruSeq DNA Sample Prep Kit (Illumina). Exome capture was done using the SureSelect Human All Exon 50 Mb kit (Agilent Technologies). Paired-end sequencing was done on an Illumina HiSeq 2000 (Illumina), generating 100-base reads. The sequences were aligned with the human genome reference sequence (hg19 build), with the Burrows-Wheeler aligner (v.0.7.12). Downstream processing was done using the Genome Analysis Toolkit (GATK, v.3.4), SAMtools (v.1.0) and Picard Tools (http://picard.sourceforge.net; v.1.92). Substitution and insertion or deletion (indel) calls were made using a GATK unified genotyper and GATK IndelGenotyperV2, respectively. All calls with a Phred-scaled single-nucleotide polymorphism quality of up to and including 20 and a read coverage of 2 or less were filtered out. All variants were annotated with annotation software developed in-house.

### Mutation enrichment analysis of whole-exome sequencing data

We performed an enrichment analysis for homozygous non-synonymous and essential splice-site variants on our cohort of 319 HSE patients and 8,500 people with other infectious diseases (controls). We considered non-synonymous and essential splice-site variants with a MAF below 0.01. We searched for all variants present in the homozygous state in HSE patients, the 8,500 controls and the gnomAD database (v.2.1.1). We compared the proportions of patients and controls homozygous for variants in a logistic regression model, accounting for the ethnic heterogeneity of the cohorts by including the first five principal components of the principal component analysis (PCA). The PCA for ethnic heterogeneity was done using PLINK (v.1.9) on whole-exome sequencing and whole-genome sequence data, with the 1000 Genomes Project phase 3 public database as a reference, using more than 15,000 exonic variants with a MAF of more than 0.01 and a call rate greater than 0.99. We also performed Fisher’s exact test to compare the frequency of homozygous variants of *TMEFF1* between our HSE cohort and gnomAD.

### Sanger sequencing of genomic DNA

Genomic DNA samples from controls, the two patients and their parents were used as a template for the amplification of regions with 300–600 base pairs (bp) encompassing the mutation by PCR with site-specific oligonucleotide primers. The PCR products were purified by ultracentrifugation through Sephadex G-50 Superfine resin (Amersham-Pharmacia-Biotech) and sequenced with the Big Dye Terminator Cycle Sequencing Kit on an ABI Prism 3700 Genetic Analyzer (Applied Biosystems). The Applied Biosystems Foundation Data Collection (v.3.0) was used for the collection of Sanger sequencing data. SnapGene software (v.7) was used for sequence analysis.

### Viral serological study by enzyme-linked immunosorbent assay and VirScan

Plasma was collected from P1 and P2 eight and ten years, respectively, after the HSE episode. Serological tests for a group of common viruses used conventional enzyme-linked immunosorbent assays to measure the antiviral IgG antibodies against various viruses, using the Liaison XL platform (Diasorin). The results were obtained in the form of relative light units but are presented here as units per millilitre of serum used. VirScan assays were used to assess antibody responses to a broader range of viruses or other pathogens on the basis of stringent cut-offs defined according to the number of non-overlapping enriched peptides, or seropositivity against a single recurrently targeted diagnostic peptide present in at least 30% of known positive samples, as previously described^34^. The integers on the VirScan heat map represent the number of significant non-overlapping enriched peptides of low homology for each virus recognized by the antiviral antibodies in each sample. Despite its high sensitivity and specificity, VirScan cannot detect epitopes that require post-translational modifications, or discontinuous sequences on protein fragments more than 56 amino acids in length. VirScan may also be less specific than certain nucleic acid-based tests in differentiating closely related virus strains^34^.

### Plasmids

The *TMEFF1* (accession number Q8IYR6) cDNA was inserted into the pDONOR cloning vector. Site-directed mutagenesis was used to obtain the mutant constructs indicated. All TMEFF1 and NECTIN-1 (Delta canonical isoform Q15223-1) constructs were then inserted into pTRIP for overexpression studies. For FRET assays^61^, CFP3A was inserted into the N-terminal region of TMEFF1 after the signal peptide, whereas pSYFP2-C1 was inserted into the N-terminal region of NECTIN-1, HVEM and PILRa after the signal peptide. The pSCFP3A-C1 and pSYFP2-C1 plasmids used for cloning were gifts from D. Gadella (Addgene plasmids #22879 and #22878). All primers used for subcloning or the production of truncated protein constructs were generated using SnapGene software (v.7). For lentiviral vector production, envelope plasmid pCMV-VSV-G, packaging plasmid PsPAX2 and transfer plasmid pTRIP were used. All constructs were resequenced to ensure that no adventitious mutations were generated during the cloning process.

### Western blotting

HEK293T cells were lysed in NP-40 lysis buffer (280 mM NaCl, 50 mM Tris, pH 8, 0.2 mM EDTA, 2 mM EGTA, 10% glycerol, 0.5% NP-40) supplemented with 1 mM DTT, PhosSTOP (Roche) and cOmplete Protease Inhibitor Cocktail (Roche). The protein lysate was subjected to SDS–PAGE and the resulting bands were transferred to a nitrocellulose membrane, which was probed with unconjugated primary and secondary antibodies. An anti-GAPDH antibody (Santa Cruz Biotechnology) was used as a loading control. We used an antibody recognizing the N terminus of the TMEFF1 protein at a dilution of 1:500 (Santa Cruz Biotechnology, B4, sc-393457). This antibody, the anti-Flag (Sigma-Aldrich, A8592; 1:1,000 dilution) and anti-Myc (Cell Signaling Technology, 2040; 1:1,000 dilution) antibodies and GAPDH (sc-47724, Santa Cruz Biotechnology; 1:5,000 dilution) were purchased from commercial suppliers. The membrane was incubated overnight at 4 °C with the primary antibodies. SuperSignal West Pico Chemiluminescent substrate (Thermo Fisher) was used to visualize HRP activity, and this signal was detected with an Amersham Imager 600 (GE Life Sciences).

### Co-immunoprecipitation

Two million HEK293T cells were used to seed 6-cm dishes overnight. They were cotransfected with 2 µg empty vector or TMEFF1 plasmid and 2 µg of a Flag-tagged plasmid encoding an HSV-1 receptor (NECTIN-1, PILRa or HVEM) or an HSV-1 glycoprotein (gB, gD, gH, gL or gC). After 36–48 h, the cells were collected, washed with ice-cold PBS and lysed in IP buffer containing 1.0% (vol/vol) Triton X-100, 10% (vol/vol) glycerol, 100 mM Tris-HCl, pH 7.4, 1 mM EDTA, 75 mM NaCl and a protease-inhibitor cocktail (Roche). The crude whole-cell lysates were centrifuged and the supernatants were collected and incubated overnight at 4 °C with protein G magnetic beads (Bio-Rad) plus 2 µg anti-TMEFF1 antibodies (Santa Cruz Biotechnology, B4, sc-393457) or mouse IgG isotype control (Santa Cruz Biotechnology, sc-2025). The NECTIN-1 endogenous IP assays used 2 µg anti-NECTIN-1 antibodies (Thermo Fisher, 37-5900). For other co-IP assays, anti-Flag M2 affinity agarose beads (Sigma-Aldrich, F2426) or anti-Myc agarose beads (Sigma-Aldrich, A7470) were used in accordance with the manufacturer’s instructions. The beads were then washed three times with IP buffer and the corresponding immunoprecipitates were eluted with 1% SDS in protein loading buffer at 95 °C for 10 min. For immunoblotting, whole-cell lysates and immunoprecipitates were subjected to SDS–PAGE. The resulting bands were transferred onto PVDF membranes, which were then probed with anti-TMEFF1 (Santa Cruz Biotechnology, B4, sc-393457) at a dilution of 1:500, anti-Nectin-1 (Thermo Fisher, 37–5900; 1:250 dilution), anti-Flag M2 HRP (Sigma-Aldrich, A8592; 1:1,000 dilution), anti-c-Myc (9E10) HRP (Cell Signaling Technology, 2272 S) and anti-GAPDH mouse monoclonal (Proteintech, HRP-60004; 1:5,000 dilution) antibodies.

### Immunostaining and confocal imaging

Cortical neurons were plated on MatTek 35 mm #1.5 glass coverslips (MatTek Corporation, P35G-1.5-14-C) at a density of 1.5 × 10^5^ per cm^2^. The endogenous TMEFF1 in cortical neurons was stained by overnight incubation with rabbit polyclonal anti-TMEFF1 antibody (Biorbyt, orb325220) at 1:1,000 dilution, before counterstaining with goat anti-rabbit AF488 secondary antibody (Invitrogen, A11034; 1:500 dilution) for 1 h. Endogenous NECTIN-1 in HEK293T cells was stained by overnight incubation with mouse monoclonal anti-NECTIN-1 antibody (Novus Biologicals, NBP2-54643-0.1 mg) at 1:500 dilution, before counterstaining with goat anti-mouse AF647 polyclonal antibody (BioLegend, poly4053, 405322) at 1:500 dilution for 1 h. Cell surface staining was performed with the MemBrite Fix-ST 755/777 cell-surface staining kit (Biotium, 30104-T) or WGA Alexa Fluor Plus 770 (Thermo Fisher, W56134) according to the manufacturer’s protocol. AT-rich chromosomal DNA was stained with DAPI (Thermo Fisher, 62248) according to the manufacturer’s protocol. Cortical neurons were then imaged with an inverted LSM 980 laser scanning confocal microscope (Zeiss) equipped with a 60× 1.4 NA oil objective lens, 34 spectral detection channels (using a GaAsP PMT detector, two multialkali PMTs, one NIR GaAS PMT, one NIR GaAsP PMT and one Airyscan detector) and an Axiocan705 monochrome camera using Zeiss ZEN Blue acquisition software (v.3.5).

### HSV-1 gD cell-surface binding assay

WT or *TMEFF1*-KO HEK293T cells stably expressing NECTIN-1 were plated in 48-well plates at a density of 5 × 10^4^ cells per well and treated with recombinant HSV-1 gD with a His tag (ACRO Biosystems Inc, GLD-V52H3) for 150 min at 37 °C. Cells were collected and fixed by incubation with 4% PFA for 15 min and blocked by incubation with 6% BSA in PBS for 1 h. Cells were then stained with PE-conjugated mouse anti-NECTIN-1 (BioLegend, R1.302, 340404) at a dilution of 1:2,000 and APC-conjugated mouse anti-His-Tag (BioLegend, J095G46, 362605) antibodies for 30 min. Cells were then washed twice with PBS and analysed by flow cytometry.

### Flow cytometry

For assessments of the expression of TMEFF1 at the cell surface, cells were plated in six-well plates at a density of 5 × 10^5^ cells per well and surface-stained with purified mouse anti-TMEFF1 antibody (Santa Cruz Biotechnology, B4, sc-393457) at a dilution of 1:1,000. They were then washed three times with PBS + 1% FCS and incubated for 30 min with AF488-conjugated anti-mouse antibody diluted 1:1,000 in PBS + 1% FCS (Invitrogen, A11001). The cells were then washed twice with PBS + 1% FCS and analysed by flow cytometry. Samples were acquired on a Gallios flow cytometer (Beckman Coulter) and the results were analysed using FlowJo software (Tree Star).

For assessments of the expression of endogenous NECTIN-1 at the cell surface, HEK293T cells were plated in 48-well plates at a density of 5 × 10^4^ cells per well and surface-stained with PE-conjugated mouse anti-NECTIN-1 (BioLegend, R1.302, #340404) at a dilution of 1:2,000 for 1 h. Cells were washed twice with PBS and analysed by flow cytometry. Samples were acquired on an LSRII flow cytometer (BD Biosciences) and the results were analysed using FlowJo software (Tree Star).

### In vitro cell stimulation

SV40 fibroblasts or hPSC-derived cortical neurons were used to coat a six-well plate at a density of 1.5 × 10^5^ cells per cm^2^. For the assessment of TLR3-associated responses, the cells were stimulated with the TLR3 agonist poly(I:C) (Tocris, 4287; a mixture of low-molecular-weight (250–1,000 bp) and high-molecular-weight (more than 1,000 bp)) at a concentration of 25 µg ml^−1^ and collected for assessment by RT–qPCR at various time points (2 h, 4 h and 6 h). For the assessment of HSV-1-induced responses, cells were infected with HSV-1 (MOI 1) and collected for assessment by RT–qPCR 24 h after infection. To assess type I interferon responses, cells were stimulated with either IFNα_2b_ (Schering, NDC-0085-0120-02; 1,000 units per ml) or IFNβ (PBL Assay Science 11415-1; 100 units per ml) and collected for assessment by RT–qPCR 8 h after stimulation.

### RT–qPCR

RNA was isolated from commercially available human tissues (Clonetech, 636643), peripheral blood mononuclear cells, fibroblasts, hPSC-derived cortical neurons, HeLa or HEK293T cells with and without plasmid transfection using the Quick-RNA MicroPrep Kit and Zymo-Spin IC Columns (R1051, Zymo Research), according to the manufacturer’s protocol. We extracted mRNA from the cells with a cell-to-CT kit (AM1729, Thermo Fisher), according to the manufacturer’s instructions. RT–qPCR was performed on an Applied Biosystems 7500 Fast Real-Time PCR System with Applied Biosystems TaqMan assays for TMEFF1 (Hs00902905_m1, spanning exons 1–2; Hs00186495_m1, spanning exons 9–10), NECTIN-1 (Hs01591978_m1), HVEM (Tnfrsf14) (Hs00998605_g1), PILRa (Hs00956112_m1) and the β-glucuronidase (GUS, #4310888E) housekeeping gene for normalization. Results were analysed using Applied Biosystems 7500 software (v.2.0.6) and are expressed according to the ΔΔCt method, as described in the manufacturer’s kit.

### Bulk RNA-seq and analysis

RNA was extracted from hPSC-derived cortical neurons using the Quick-RNA MicroPre Kit (R1051, Zymo Research). RNA-seq libraries were prepared with the Illumina RiboZero TruSeq Stranded Total RNA Library Prep Kit (Illumina) and RNA-seq was done on the Illumina NovaSeq platform, with a read length of 100 bp and a read depth of around 40 million reads. All samples were sequenced in technical duplicates. All FASTQ files passed quality control and were aligned with the GRCh38 reference genome with STAR (2.6.1d). Gene-level features were quantified using featureCounts v.1.6.0 based on GRCh38 gene annotation. Count data were normalized using counts per million in the EdgeR package (v.3.40.2)^62^, dimension-reduced through PCA and subjected to heat-map analysis using ComplexHeatmap (v.2.14.0)^63^. Differential expression analysis was done using DESeq2 (v.1.38.3)^64^. For the isoform-level analysis of *NECTIN1*, RNA-seq FASTQ files were pseudo-aligned with transcriptome indices (Ensembl release 110) with Kallisto (v.0.48.0)^65^. Transcriptomics-level features were quantified and normalized as transcripts per million. Duplicates were studied for each set of conditions and mean gene expression levels were used for subsequent analyses.

### hPSC culture and characterization

Patient-specific iPSCs were obtained by reprogramming the patients’ primary fibroblasts by infection with the non-integrating CytoTune Sendai viral vector kit (Life Technologies). Human embryonic stem cell (hESC) or iPSC (together referred to as hPSC) cultures were maintained in Essential 8 medium (Life Technologies, A1517001). We used one healthy control hESC line (H9, which we were allowed to use following the Materials Transfer Agreement from WiCell for the described experiments), one healthy control iPSC line (BJ1) and iPSCs from other HSE patients with autosomal recessive TLR3 or IFNAR1 deficiencies (*TLR3*^−/−^ and *IFNAR1*^−/−^) that were made available from our previous studies^22,24^. We also used two gene-edited *TMEFF1*-KO iPSC lines and two patient-specific *TMEFF1*-mutated iPSC lines that were made available through this study. The newly derived iPSC lines were verified for the elimination of transgene expression before biobanking and experimental use^24^. Patient-specific *TMEFF1* mutations were confirmed by the Sanger sequencing of genomic DNA extracted from patient iPSC lines. All hPSCs were karyotyped to ensure that the genome was intact.

### Differentiation of cortical neurons from hPSCs

Cortical neurons were differentiated from hPSCs grown in E8 essential medium on 10-cm plates coated with VTN-N (Thermo Fisher). Cells were maintained at 37 °C in an atmosphere containing 5% CO_2_. The hPSCs were differentiated into cortical neurons according to a published protocol^37^. In brief, hPSCs were dissociated by Accutase (Innovative Cell Technologies, AT-104) treatment to obtain a single-cell suspension, which was plated at a density of 300,000 cells per cm^2^ in Essential 8 medium supplemented with ROCK inhibitor (Y-27632 dihydrochloride, 10 µM; Tocris, 1254) on Matrigel-coated plates. Cells were cultured in Essential 8 medium containing LDN193189 (100 nM) (Stemgent, 04-0074) and SB431542 (10 µM) (STEMCELL Technologies, 72234) for 10 days, with the addition of XAV939 (2 µM; Tocris, 3748/10) for the first three days of differentiation. Cells were allowed to differentiate for 11–20 days in N2-based medium (Gibco, 17502048) supplemented 1:1,000 with B27 (Gibco, 12587010) to promote the development of neural progenitor cells. These cells were then dissociated and replated on polyornithine/fibronectin/laminin-coated plates and maintained in Neurobasal medium supplemented with BDNF (R&D Systems, 248-BD), ascorbic acid (L-AA, Sigma-Aldrich, A4034), GDNF (Peprotech, 450-10), cAMP (Sigma-Aldrich, D0627), l-glutamine (Gibco, 35050061) and B27 (Gibco, 12587010) to promote neuronal differentiation and maturation. All experiments were performed on hPSC-derived cortical neurons at DIV 50.

### TOPO cloning and sequencing of cDNAs from patient cells

Total RNA was extracted using the RNeasy mini kit (Qiagen) from SV40-transformed fibroblasts and hPSC-derived cortical neurons. The RNA was reverse-transcribed using the SuperScript III First-Strand Synthesis System (Thermo Fisher), according to the manufacturer’s instructions. PCR was performed with 2× Taq PCR master mix (APExBIO) and the *TMEFF1* primers (forward, 5′-GCCTTGCCCTGAAAACCTCA-3′; reverse, 5′- GTTCATGCGATAGGCAGTGTC-3′). The PCR products were inserted into the pCR2.1-TOPO vector (Life Technologies) and used to transform Stellar competent cells (Takara Bio). At least 100 colonies per subject were picked for P1 and a healthy control. Finally, we performed PCR on these colonies and sequenced them with *TMEFF1* primers (forward, 5′-GTAAAACGACGGCCAG-3′; reverse: 5′-CAGGAAACAGCTATGAC-3′).

### CRISPR–Cas9-mediated gene knockout

Gene-editing experiments were performed as previously described^36^. In brief, guide RNA sequences were generated using the CRISPR design tool (http://crispr.mit.edu/). The Cas9 target site for human *TMEFF1* is 5′-GATGAGTCATCATGTAAATA-3′. The guide RNA was inserted into the pSpCas9(BB)-2A-EGFP vector, which was then used to transfect BJ1 iPSC cells in the presence of Lipofectamine LTX (Invitrogen); 24 h later, EGFP signals were sorted by flow cytometry on cells in a 96-well plate. The single-cell clones were genotyped by Sanger sequencing. For BJ1 *TMEFF1*-KO genotyping, the following primers were used in this study (forward, 5′-GCAGGATTCTGTTTGGGGAATAC-3′; reverse, 5′-CCTCTCAATTCCATGCAAGCAG-3′).

Gene editing was performed using HEK293T or HeLa cells by electroporation with the Synthego CRISPR Gene Knockout Kit v.2. The Cas9 target site for human *TMEFF1* is 5′-CAGCCGGAGCGGCGCCTCAG-3′, 5′-CGCGCGTCCAACCAGCCCCC-3′ and 5′-ATGCTCTTGCCTTTGCCGCC-3′, and the target site for human *NECTIN1* is 5′-GCAGGAATTCCACACGCTCG-3′, 5′-GTAGATGGCCACGTTCTGCT-3′ and 5′-GTGATCTTCACGCTGGGAAG-3′. Bulk or single-cell clones were genotyped by Sanger sequencing. For HEK293T and HeLa *TMEFF1*-KO genotyping, the following primers were used: forward, 5′-CACAAAGGGAAGGCGAGGA-3’; reverse, 5′-CCACGACGGGGTCTTTCC-3′. For HEK293T and HeLa *NECTIN1*-KO genotyping, the following primers were used: forward, 5′-TCTGGATGAACAGGGAGGGG-3′; reverse, 5′-AACTGTGTGGGTGGGGG-3′.

### HSV-1 entry assay

We cultured iPSC-derived cortical neurons as described above. The delivery of β-lactamase to cells through HSV-1 entry was assessed in the CCF2 assay, as previously described^47^. The medium was replaced with 0.6 µl CCF2-AM, 5.4 µl solution B, 79 µl solution C (CCF2-AM Live-Blazer dye solution; Invitrogen, K1032), 15 µl probenecid and 500 µl of the initial medium. Cells were incubated for 40 min at 37 °C under an atmosphere containing 5% CO_2_ to load the cytosol with the CCF2 substrate. The cells were then washed three times with medium and infected at a MOI of 100 with HSV-1 in which β-lactamase was fused to the C terminus of pUL47 (HSVF-GS#6389) or WT HSV-1 (GS#2695) as a negative control. One hour after infection, the cells were imaged using an Olympus IX-70 inverted microscope equipped with a 40×, 1.3 NA oil objective lens, DAPI (excitation filter 381–399 nm, emission filter 435/48 nm) and FITC (emission filter 525/50 nm) filter sets and a pco.edge scientific complementary metal–oxide–semiconductor (sCMOS) camera using the SoftWoRx acquisition software (V6.5.2). Three sequential images of a single field were captured, the first being a fluorescence image obtained with a 381–399 nm excitation filter and a 435/48 nm emission filter. The second image was obtained with a 381–399 nm excitation filter and a 525/50 nm emission filter, and the final image was a bright-field image. CCF2 cleavage was quantified by drawing a region of interest (ROI) in the centre of the cell on the bright-field image and then determining the mean fluorescence intensity in the ROI for the 435 nm and 525 nm images. Ratiometric values were calculated by dividing the mean fluorescence intensity of the 435 nm ROI by the corresponding mean fluorescence intensity of the 525 nm ROI. Ratiometric values were obtained for three independent experiments with at least 30 ROIs recorded for each set of conditions in each experiment. Each dot shown in the results represents a measurement of intracellular CCF2 cleavage from one cell. Image analysis was performed with ImageJ software (v.2.3.0/1.53f). Statistical analysis was done using GraphPad Prism 9 (v.9.2.0).

### HSV-1 nuclear translocation reporter assay in cortical neurons

The iPSC-derived cortical neurons were infected with HSV-1 fused to the immediate–early RFP reporter (HSVF-GS#3217)^66–69^. Cells were fixed with 4% paraformaldehyde in PBS 10 h after infection. Cortical neurons were stained with anti-MAP2 antibody at a dilution of 1:1,000 (Abcam, ab11267) and were then counterstained with anti-mouse IgG Alexa Fluor 488 at a dilution of 1:500 (Invitrogen, A11001) antibody. Nuclei were labelled with DAPI (Thermo Fisher, 62248). Images were captured with an Olympus IX-70 inverted microscope equipped with a 40×, 1.3 NA oil objective lens and a pco.edge scientific complementary metal–oxide semiconductor camera. A ROI was drawn at the centre of the nucleus of cortical neurons expressing MAP2, and RFP intensity was measured in it. Cells were considered to be infected if their RFP emissions were more than 3.1 times higher than the background of the field. We analysed a minimum of 50 cells per condition. Images were acquired for at least three independent experiments. Image analysis was performed with ImageJ software (v.2.3.0/1.53f). Statistical analysis was performed using GraphPad Prism 9 (v.9.2.0).

### HSV-1 nuclear translocation reporter assay in HEK293T and HeLa cells overexpressing *TMEFF1*

HEK293T and HeLa cells transiently transfected with *TMEFF1* variants were infected with HSVF-GS#3217. Cells were fixed with 4% paraformaldehyde 10 hours after infection. Cells were permeabilized with 0.1% Triton-X100 (Sigma-Aldrich) in PBS (PBST) and blocked by incubation with 6% goat serum in PBST for one hour. Cells were then stained by overnight incubation with mouse anti-TMEFF1 antibody at a dilution of 1:250 (Santa Cruz Biotechnology, B4, sc-393457) and counterstained by incubation with anti-mouse IgG Alexa Fluor 488 antibodies at a dilution of 1:500 (Invitrogen, A11001). Nuclei were labelled with DAPI (Thermo Fisher, 62248). Images were captured with an LSM 880 Airyscan NLO inverted laser scanning confocal and multiphoton microscope equipped with a 63×, 1.4 NA oil objective lens using Zeiss ZEN Black acquisition software (v.2.3 SP1). A ROI was drawn at the centre of the nucleus of cells overexpressing *TMEFF1*, and RFP intensity was measured in it.

### HSV-1 infection and quantification of viral replication

For WT HSV-1 (KOS strain, ATCC) infection, 1.75 × 10^5^ cortical neurons per well were used to seed 48-well plates. The cells were infected at an MOI of 0.001 in neuron culture medium. After 2 h, the cells were washed and 250 μl fresh medium was added to each well. Both cells and supernatants were collected at various time points after HSV-1 infection and frozen. HSV-1 titres were determined by calculating the TCID_50_ per ml on Vero cells (ATCC), as previously described^21^.

### Statistical analysis

Where applicable, results are presented as mean ± s.e.m. or median ± interquartile range. Mean values were compared between control cells and cells from the patients in one-way ANOVA with Tukey tests for multiple comparisons. Where indicated, linear mixed models were used for log-transformed relative values to account for repeated measurements. Kruskal–Wallis tests with Dunn’s test for multiple comparisons were used if the data were found to follow a non-normal distribution. Two-tailed Mann–Whitney *U* tests were used for comparisons between two groups. No blinding or randomization were used in this study. Statistical analysis was performed using SPSS 19.0 and GraphPad Prism 9 (v.9.2.0). Statistical significance is denoted as follows: NS, not significant; *P* > 0.05; *P < 0.05; **P < 0.01; ***P < 0.001; ****P < 0.0001.

### Reporting summary

Further information on research design is available in the Nature Portfolio Reporting Summary linked to this article.

## Online content

Any methods, additional references, Nature Portfolio reporting summaries, source data, extended data, supplementary information, acknowledgements, peer review information; details of author contributions and competing interests; and statements of data and code availability are available at 10.1038/s41586-024-07745-x.

### Supplementary information

Supplementary Information This file contains Supplementary Table 1 (Homozygous rare non-synonymous or essential splicing variants in patients 1 and 2), Supplementary clinical reports of patients 1 and 2 regarding their HSE episodes and Supplementary Fig. 1 (a graphical abstract representing the mechanism by which human TMEFF1 deficiency renders CNS neurons susceptible to HSV-1 and underlies HSE).

Reporting Summary

### Source data

Source Data for Figs. 1–6 and Source Data Extended Data Figs. 3–11.

## Data Availability

All data are presented in the paper, with raw values, exact statistical *P*-values and images of gels provided as Source Data. The human genome reference sequence (hg19 build) is available in the NCBI database (https://www.ncbi.nlm.nih.gov/datasets/genome/GCF_000001405.13/). For population genetics analysis, we used available data from the public database gnomAD (http://gnomad.broadinstitute.org). The raw RNA-seq data generated from this study are accessible in the NCBI database under the NCBI-SRA project PRJNA1107163 (https://www.ncbi.nlm.nih.gov/bioproject/PRJNA1107163/).
