# Supplementary material for: Human TMEFF1 is a restriction factor for herpes simplex virus in the brain
Source: Nature. 2024 Jul 24;632(8024):390–400. doi: 10.1038/s41586-024-07745-x (PMC11306101; doi:10.1038/s41586-024-07745-x)
Supplement: Supplementary file 1 — This file contains Supplementary Table 1 (Homozygous rare non-synonymous or essential splicing variants in patients 1 and 2), Supplementary clinical reports of patients 1 and 2 regarding their HSE episodes and Supplementary Fig. 1 (a graphical abstract representing the mechanism by which human TMEFF1 deficiency renders CNS neurons susceptible to HSV-1 and underlies HSE). [file 41586_2024_7745_MOESM1_ESM.pdf]

nature portfolio

https://doi.org/10.1038/s41586-024-07745-x

Supplementary information

Human TMEFF1 is a restriction factor for herpes simplex virus in the brain

In the format provided by the
authors and unedited

nature portfolio

https://doi.org/10.1038/s41586-024-07745-x

Supplementary information

Human TMEFF1 is a restriction factor for herpes simplex virus in the brain

In the format provided by the authors and unedited

Supplementary information guide

Supplementary Table 1.
Homozygous rare nonsynonymous or essential-splicing variants in patients 1 and 2 (PDF format).

Supplementary Clinical Reports.
Clinical reports of patient 1 and 2 about their HSE episodes (PDF format).

Supplementary Figure 1.
Graphical abstract representing the mechanism by which human TMEFF1 deficiency renders CNS neurons susceptible to HSV-1 and underlies HSE (PDF format).

Table S1. Homozygous rare nonsynonymous or essential-splicing variants in patients 1 and 2

Pt #	Gene	GDI	Variation type	Change	Zygosity	MAF (gnomAD)	CADD
1	*DEPTOR*	9.83	missense	p.Gly5Asp	hom	3.82E-04	23
	IFT81	4.07	missense	p.Leu420Phe	hom	0.00494474	5.42
	PITRM1	12.05	missense	p.Leu884Phe	hom	0.00949226	25.7
	RBM11	5.45	missense	p.Asn109Lys	hom	0.00688205	13.88
	TMEFF1	0.63	missense	p.Pro44Ala	hom	1.3E-03	11.2
	ZNF717	2.86	indel-frameshift	p.Ser321fs	hom	0.00426959	18.33
	KCNN3	4.28	indel-inframe	p.Gln77_Gln80dup	hom	-	11.47
	GABRR1	8.81	missense	p.Lys375Asn	hom	-	29.8
	C11orf80	2.35	indel-inframe	p.Ala34dup	hom	-	7.4
	P2RX4	7.9	missense	p.Arg33Ser	hom	-	34
	ATXN3	12.92	indel-inframe	p.Gln297_Gln305dup	hom	-	5.41
	MROH8	-	stop-gained	p.Ser57fs	hom	-	25.4
2	*TMEFF1*	0.63	essential_splicing	-	hom	1.8E-05	23.5
	OR13C8	3.42	stop-gained	p.Ser9*	hom	0.0088552	26.4
	ABCA1	12.16	missense	p.Lys776Asn	hom	0.00146487	27.6
	SPTAN1	2.21	missense	p.Ala504Val	hom	8.60E-04	24.5
	MAMDC4	5.19	missense	p.Pro966Leu	hom	0.00235879	31
	CCDC73	2.63	missense	p.Asn6Ser	hom	0.00506563	3.52
	SLCO1B3	-	missense	p.Thr414Ile	hom	1.28E-04	12.13
	TLN2	8.68	missense	p.Thr135Ile	hom	5.42E-04	21.8
	DENND4A	3.95	missense	p.Val877Ile	hom	3.19E-05	28.5
	BLM	5.93	missense	p.Thr298Met	hom	0.00579876	9.68
	RGMA	5.53	missense	p.Arg441Trp	hom	0.00325442	17.27
	CENPBD1	3.9	missense	p.Cys150Tyr	hom	0.00799516	20.5
	PALM	1.66	missense	p.Glu349Val	hom	7.67E-04	25.6
	CRYZL1	1.89	missense	p.Ile220Val	hom	0.00617952	23.4
	SRPX2	1.41	missense	p.Ser150Phe	hom	9.08E-05	23.1
	GPR50	6.34	missense	p.His552Gln	hom	3.21E-04	17.13
	HCFC1	1.74	missense	p.Thr958Ala	hom	7.34E-04	25.3
	KCNN3	4.28	indel-inframe	p.Gln75_Gln80dup	hom	-	10.95
	LUZP6	1.23	missense	p.Pro18His	hom	-	15.06
	NOL8	5.53	missense	p.Thr950Ala	hom	-	25.2
	TBC1D13	0.8	missense	p.Cys204Arg	hom	-	8.48
	ATL3	5.28	stop-gained	p.Arg6*	hom	-	36
	KLHL33	11.6	missense	p.Ser125Asn	hom	-	6.49
	IMP3	1.31	missense	p.Val142Ala	hom	-	23
	FADS6	5.62	indel-inframe	p.Pro6ins_18aa	hom	-	6.6
	RNF126	4.37	missense	p.Thr219Ala	hom	-	16.09
	APC2	9.57	missense	p.Pro1468Leu	hom	-	22.9
	PRMT2	0.68	missense	p.Leu283Ile	hom	-	4.22
	MAGEB4	0.37	missense	p.Asp48Tyr	hom	-	20.1
	PLXNB3	5.36	missense	p.Val1534Leu	hom	-	14.85

Note: homozygous nonsynonymous or essential-splicing variants (MAF < 0.01 in gnomAD (V2.1.1), CADD > MSC 99%) were found in 12 genes (GDI < 13.83) in the exome of P1 and 30 genes in the exome of P2. MAF: minor allele frequency. GDI: gene damaging index (https://lab.rockefeller.edu/casanova/GDI)[29]. CADD: combined annotation-dependent depletion score (https://cadd.gs.washington.edu/)[27].

Supplementary clinical reports

Patient 1(P1) is a 19-year-old girl born to non-consanguineous parents of Algerian and Moroccan origin living in France. She was first admitted to hospital at the age of two and a half years, for high fever and coma. Cerebral MRI showed large cortical lesions in the left temporal region. PCR on CSF was negative for herpes simplex virus, and serological tests for antibodies against various viruses yielded negative results. Laboratory testing of the CSF sample taken at day 1 of hospitalization showed glucose levels at ~4.8 g/l, chloride levels at ~107 mmol/l, protein levels at ~0.23 g/l, 4 leukocytes/mm^3, 12 red blood cells/mm^3. Serological tests for other viruses, including Epstein-Barr virus, picornavirus, parvovirus and varicella zoster virus, yielded negative results. A diagnosis of herpes simplex virus encephalitis (HSE) was suspected, and the patient was treated with intravenous acyclovir. She gradually recovered and was discharged from the hospital 10 days later, with neurological sequelae presenting as right facial paralysis and difficulty speaking. About three weeks later, she was re-admitted to hospital, due to a relapse of HSE resulting in a loss of consciousness and abnormal movement of the right leg. A CSF sample taken at admission revealed high protein levels, at ~1.60 g/l, and positivity for anti-HSV-1 IgG antibody with an IgG/albumin ratio of ~1.45. PCR on CSF for herpes simplex virus was again negative, but serological tests for anti-HSV-1 antibodies became highly positive for IgG (>320 U/ml) and weakly positive for IgM. The patient was treated in the ICU for three days before being discharged from hospital. She has since suffered severe neurological sequelae, presenting as frequent spasms and epilepsy. No other severe infectious diseases or other remarkable medical history have been reported for this patient or any of her relatives.

P2 is a 19-year-old boy born to consanguineous parents of Turkish origin living in Turkey. He was healthy until his first admission to hospital at the age of five years following three days of seizures and visual hallucinations. PCR on CSF for HSV was negative and viral serological tests were not performed. Brain MRI revealed large cortical lesions in the temporo-occipital regions consistent with HSE. The patient was treated with intravenous acyclovir and recovered 10 days later. He was discharged from hospital with neurological sequelae presenting as frequent

fractional epilepsy. No other severe infectious diseases or other remarkable medical history have been reported for this patient or his relatives.

Supplementary Figure 1

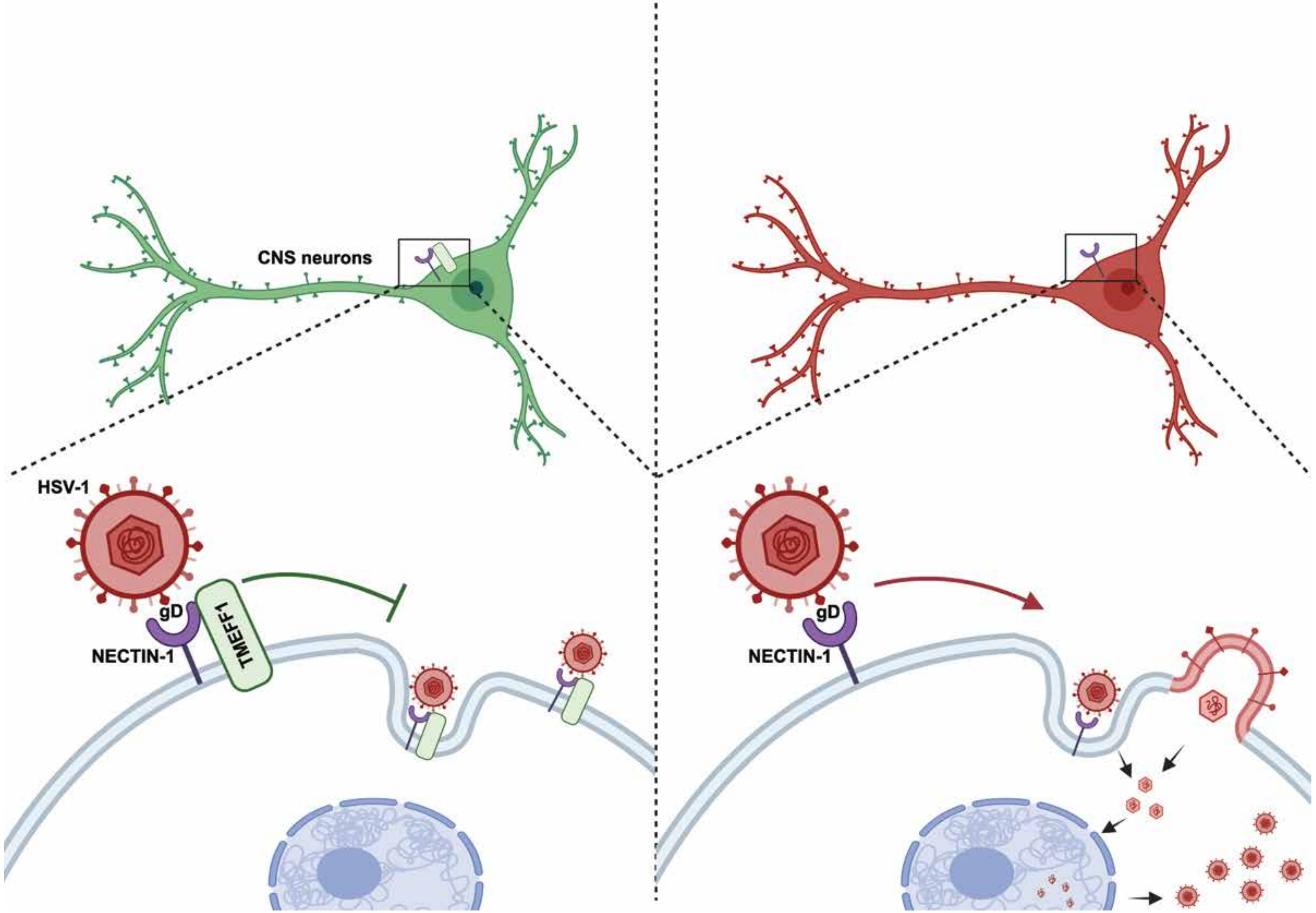

Supplementary Fig. 1: Graphical abstract representing the mechanism by which human TMEFF1 deficiency renders CNS neurons susceptible to HSV-1 and underlies HSE.
In this model, TMEFF1 interacts with the HSV-1 receptor NECTIN-1 on the cell surface of CNS cortical neurons, impairing HSV-1 glycoprotein D (gD)-human NECTIN-1-mediated virus-cell membrane fusion and viral entry. Genetic TMEFF1 deficiency allows HSV-1 to enter cortical neurons rapidly; this is followed by enhanced HSV-1 translocation to the nucleus and replication, underlying HSE. This graphical abstract was created with the BioRender app (biorender.com).
