# Supplementary material for: Human TMEFF1 is a restriction factor for herpes simplex virus in the brain
Source: Nature. 2024 Jul 24;632(8024):390–400. doi: 10.1038/s41586-024-07745-x (PMC11306101; doi:10.1038/s41586-024-07745-x)
Supplement: Supplementary file 2 — Reporting Summary [file 41586_2024_7745_MOESM2_ESM.pdf]

nature portfolio

Corresponding author(s): Shen-Ying Zhang

Last updated by author(s): Jun 2, 2024

Reporting Summary

Nature Portfolio wishes to improve the reproducibility of the work that we publish. This form provides structure for consistency and transparency in reporting. For further information on Nature Portfolio policies, see our Editorial Policies and the Editorial Policy Checklist.

Statistics

For all statistical analyses, confirm that the following items are present in the figure legend, table legend, main text, or Methods section.

n/a	Confirmed	
☐	☒	The exact sample size (*n*) for each experimental group/condition, given as a discrete number and unit of measurement
☐	☒	A statement on whether measurements were taken from distinct samples or whether the same sample was measured repeatedly
☐	☒	The statistical test(s) used AND whether they are one- or two-sided<br>*Only common tests should be described solely by name; describe more complex techniques in the Methods section.*
☒	☐	A description of all covariates tested
☐	☒	A description of any assumptions or corrections, such as tests of normality and adjustment for multiple comparisons
☐	☒	A full description of the statistical parameters including central tendency (e.g. means) or other basic estimates (e.g. regression coefficient) AND variation (e.g. standard deviation) or associated estimates of uncertainty (e.g. confidence intervals)
☐	☒	For null hypothesis testing, the test statistic (e.g. *F*, *t*, *r*) with confidence intervals, effect sizes, degrees of freedom and *P* value noted<br>*Give P values as exact values whenever suitable.*
☒	☐	For Bayesian analysis, information on the choice of priors and Markov chain Monte Carlo settings
☒	☐	For hierarchical and complex designs, identification of the appropriate level for tests and full reporting of outcomes
☒	☐	Estimates of effect sizes (e.g. Cohen's *d*, Pearson's *r*), indicating how they were calculated

Our web collection on statistics for biologists contains articles on many of the points above.

Software and code

Policy information about availability of computer code

Data collection	AB 7500 Software V2.0.6 was used for qPCR data collection from the Applied Biosystems 7500 Fast Real-Time PCR System.<br>Amersham Imager 600 and related software was used to collect western-blot imaging data.<br>AB Foundation Data Collection Version 3.0 was used for Sanger sequencing data collection from the Applied Biosystems 3730 DNA Analyzer.<br>Beckman Coulter Gallios and BD LSRII cytometer were used to collect flow cytometry data.<br>Zeiss LSM 880 Airyscan NLO inverted laser scanning confocal and multiphoton microscope, with Zeiss Zen Black acquisition software (V2.3 SP1), was used to collect confocal imaging data.<br>Zeiss LSM 980 inverted laser scanning confocal microscope, with Zeiss Zen Blue acquisition software (V3.5), was used to collect confocal imaging data.<br>Olympus IX-70 inverted microscope, with SoftWoRx acquisition software (V6.5.2), was used to collect immunofluorescence imaging data.<br>Illumina HiSeq 2000 was used to perform Whole-exome sequencing.<br>Illumina NovaSeq was used to perform RNA-sequencing.
Data analysis	SPSS 19.0 and GraphPad Prism 9.2.0 were used for data analysis in general.<br>ImageJ V2.3.0/1.53f was used to perform image analysis.<br>Burrows-Wheeler aligner was used for aligning whole-exome sequencing data with the human genome reference sequence (hg19 build).<br>Genome Analysis Toolkit (GATK), SAM tools and Picard Tools (http://picard.sourceforge.net) were used for whole exome sequencing data analysis.<br>GATK Unified Genotyper and GATK IndelGenotyperV2 were used to perform substitution and Indel calls, respectively.<br>PLINK (v1.9) was used to perform PCA for ethnic heterogeneity on WES and WGS data, with the 1000 Genomes (1kG) Project phase 3 public database as reference.<br>STAR (v2.6.1d) was used for alignment of RNA sequencing FASTQ files with GRCh38 reference genome.

April 2023

FeatureCounts (v1.6.0) was used to quantify Gene-level features.
EdgeR package (V3.40.2) was used to normalize count data through "counts per million (CPM)" and dimensionally reduce count data through Principal component analysis (PCA).
ComplexHeatmap (V2.14.0) was used to perform heatmap analysis on RNA-seq data.
DEseq2 (V1.38.3) was used to perform differential expression analysis.
Kallisto (V0.48.0) was used to pseudo-align transcriptome indices with transcriptome indices (Ensembl release 110).
FlowJo (V10.9.0, Tree Star) was used to analyze flow cytometry data.

For manuscripts utilizing custom algorithms or software that are central to the research but not yet described in published literature, software must be made available to editors and reviewers. We strongly encourage code deposition in a community repository (e.g. GitHub). See the Nature Portfolio guidelines for submitting code & software for further information.

Data

Policy information about availability of data

All manuscripts must include a data availability statement. This statement should provide the following information, where applicable:

- Accession codes, unique identifiers, or web links for publicly available datasets
- A description of any restrictions on data availability
- For clinical datasets or third party data, please ensure that the statement adheres to our policy

All data are presented in the manuscript, with raw values, exact statistical p-values and images of gels provided as Source Data. Human genome reference sequence (hg19 build) is available in NCBI database (https://www.ncbi.nlm.nih.gov/datasets/genome/GCF_000001405.13/). For population genetics analysis, we used available data from the public database gnomAD (http://gnomad.broadinstitute.org). The raw RNA-seq data generated from this study are accessible in the NCBI database under the NCBI-SRA project PRJNA1107163.

Research involving human participants, their data, or biological material

Policy information about studies with human participants or human data. See also policy information about sex, gender (identity/presentation), and sexual orientation and race, ethnicity and racism.

Reporting on sex and gender	Study was conducted on human patient-specific mutations in TMEFF1 under an autosomal recessive inheritance model. There are no reporting on sex and gender effects in this study
Reporting on race, ethnicity, or other socially relevant groupings	The 2 patients (1 male and 1 female) that we studied are of different origins and came from different countries. They developed HSV-1 encephalitis at various ages (2.5 and 5 years).
Population characteristics	Study was conducted on two patients who carried rare mutations in TMEFF1 under an autosomal recessive inheritance model. The two patients (1 male and 1 female) that we studied are of different origins and came from two different countries. They developed HSV-1 encephalitis at various ages (2.5 years and 5 years)
Recruitment	The two patients described in this study were living in and followed up in their countries of origin. Informed consent was obtained in the home country of each patient, in accordance with local regulations and the requirements for institutional review board (IRB) approval. No self-selection bias is applicable. This study focused on the experimental characterization of the disease pathogenesis of Herpes simplex virus 1 encephalitis in the two patients, in order to understand the role of the TMEFF1 mutations carried by the two patients.
Ethics oversight	Informed consent was obtained in France, in accordance with local regulations and a human-subjects research protocol approved by the institutional review board (IRB) of Institut National de la Santé et de la Recherche Médicale (INSERM). Experiments were conducted in the United States and France, in accordance with local regulations and with the approval of the IRB of The Rockefeller University and INSERM, respectively, to conduct human genetic and immunological studies including those utilize hPSC-derived tissue-specific cells for research purpose. Approval was obtained from the French Ethics Committee (Comité de Protection des Personnes), the French National Agency for Medicine and Health Product Safety, and INSERM in Paris, France (protocol no. C10-13), and the Rockefeller University Institutional Review Board in New York, USA (protocol no. SHZ-0676). HPSC-related work was approved by the Tri-Institutional Stem Cell Initiative Embryonic Stem Cell Research Oversight Committee (protocol no. 2021-05-003).

Note that full information on the approval of the study protocol must also be provided in the manuscript.

Field-specific reporting

Please select the one below that is the best fit for your research. If you are not sure, read the appropriate sections before making your selection.

☒ Life sciences ☐ Behavioural & social sciences ☐ Ecological, evolutionary & environmental sciences

For a reference copy of the document with all sections, see nature.com/documents/nr-reporting-summary-flat.pdf

Life sciences study design

All studies must disclose on these points even when the disclosure is negative.

Sample size	No sample size calculation was performed. However, in our experimental design, we included 2 to 3 biological replicates for cells from healthy controls and those with TMEFF1 deficiency, and for all data shown (other than the RNAseq experiment), at least 3 independent experiments were performed.
Data exclusions	No data were excluded from the analyses.
Replication	For the RNA-Seq experiment, a single sequencing experiment was performed, using 1 library from each line included in the study. At least 2 biological replicates were included for the control and patient groups. Each library was sequenced twice (technical duplicates).<br>For all other experimental data shown, at least 3 independent experiments were performed for each data set. When applicable, means and SEM of values from 3 or more independent experiments are shown. All experimental data were reproducible between independent experiments.
Randomization	Randomization is not applicable to this study, as the study is focused on experimental investigation of the role of TMEFF1 in host defense by taking advantage of human patient-specific mutations that disrupt the localization and function of TMEFF1. Instead, at least 2 biological replicates were included for the control and patient groups, and independent experiments were repeated at least 3 times to control for covariates.
Blinding	Blinding for experimental group allocation is not applicable to this study, because: 1) no clinical trial is involved; 2) our experiments were designed to compare the HSV-1 infection and cellular responses between TMEFF1-mutated and TMEFF1 wild-type cells, hence the TMEFF1 genotype of the cells have to be confirmed for proper experimental design and data analysis.

Reporting for specific materials, systems and methods

We require information from authors about some types of materials, experimental systems and methods used in many studies. Here, indicate whether each material, system or method listed is relevant to your study. If you are not sure if a list item applies to your research, read the appropriate section before selecting a response.

Materials & experimental systems

n/a	Involved in the study	
☐	☒	Antibodies
☐	☒	Eukaryotic cell lines
☒	☐	Palaeontology and archaeology
☒	☐	Animals and other organisms
☒	☐	Clinical data
☒	☐	Dual use research of concern
☒	☐	Plants

Methods

n/a	Involved in the study	
☒	☐	ChIP-seq
☐	☒	Flow cytometry
☒	☐	MRI-based neuroimaging

Antibodies

Antibodies used	Anti-TMEFF1 mouse IgG (Santa Cruz Biotechnology, sc-393457, Lot#D1917)<br>Anti-TMEFF1 rabbit IgG (Biorbyt, orb325220, Lot#AB6245)<br>Anti-NECTIN-1 mouse IgG1 PE (BioLegend, #340404, Lot#B367757)<br>Anti-NECTIN-1 mouse IgG1 (Novus Biologicals, NBP2-54643-0.1mg, Lot#537383)<br>Anti-MAP2 mouse IgG1 (Abcam, ab11267, Lot#XA3473878)<br>Anti-Flag mouse IgG1 (Sigma-Aldrich, A8592, Lot#SLCF0816)<br>Anti-Myc mouse (Cell Signaling Technology, #2040, Lot#6)<br>Anti-Myc rabbit (Cell Signaling Technology, #2272, Lot#6)<br>Anti-His mouse (BioLegend, #362605, Lot#B369084)<br>Anti-GAPDH mouse IgG1 (0411) (Santa-Cruz Biotechnology, sc-47724, Lot #K0320)<br>Anti-mouse IgG Alexa Fluor 488 secondary (Invitrogen, A11001 Lot #2551357)<br>Anti-mouse IgG Alexa Fluor 647 secondary (Biolegend, #405322, Lot #B288681)<br>Anti-mouse IgG Biotin secondary (ThermoFisher Scientific, 13-4013-85,<br>Anti-rabbit IgG Alexa Fluor 488 secondary (ThermoFisher Scientific, A11034, Lot #2379999)<br>Anti-His-Tag APC secondary (BioLegend, J095G46, #362605, Lot#B387383)
Validation	Anti-TMEFF1 antibody (Santa Cruz Biotechnology, sc393457) has been previously reported and experimentally shown to specifically recognize TMEFF1 (Xie et. al., 2022, Int J Neuropsychopharmacol). Anti-TMEFF1 has also been experimentally validated by manufacturer to stain TMEFF1 in human heart tissue extract, T98G and HeLa whole cell lysates.<br>Anti-TMEFF1 antibody (Biorbyt, orb325220) has been experimentally validated by manufacturer to stain TMEFF1 in human heart tissue through western-blotting.<br>Anti-NECTIN-1 mouse IgG1 (BioLegend, #340404) has been experimentally validated by manufacturer to stain NECTIN-1 in human erythroleukemia cell line TF-1 through flow cytometry.<br>Anti-NECTIN-1 mouse IgG1 (Novus Biologicals, NBP2-54643-0.1mg) has been validated by manufacturer to stain NECTIN-1 in human

peripheral blood through glow cytometry.
Anti-MAP2 has been previously shown to specifically recognize MAP2 neuronal marker (Shi et. al., 2018, Nat Med)
Anti-Flag is a commonly used standardized antibody which recognizes the Flag peptide. Anti-Flag was validated by manufacturer and cited in over 60 publications.
Anti-Myc is a commonly used standardized antibody which recognizes the Myc peptide. Anti-Myc was tested by the manufacturer through Western-blotting in HEK293T cells transfected with a construct expressing Myc/DDK tagged MMP8.
Anti-His is a commonly used standardized antibody which recognizes the His peptide. Anti-His was tested by the manufacturer in CL hTMEM8A transfected P3.U1 stained with anti-His Tag (clone J095G46) APC or mouse IgG2a, κ APC isotype control.
Anti-GAPDH is a commonly used standardized antibody which recognizes the protein of a human cell house-keeping gene GAPDH. Anti-GAPDH antibody was tested in near-Infrared western blot analysis of GAPDH expression in Jurkat, MOLT-4, HeLa, K-562, BJAB and IMR-32 whole cell lysates by the manufacturer. Product was cited in 3,124 publications.

Eukaryotic cell lines

Policy information about cell lines and Sex and Gender in Research

Cell line source(s)

HEK293T cells, from ATCC.
HeLa cells, from ATCC.
Vero cells, from ATCC.
hES cells H9 line, from the WiCell Research Institute.
iPS cells parental BJ1 line and two gene-edited TMEFF1 KO lines, originated from the Studer laboratory at Sloan-Kettering Institute for Cancer Research.
Two healthy control SV40-fibroblast cell lines, originated from the Laboratory of Human Genetics of Infectious Diseases at Imagine Institute.

Authentication

HEK293T cells, HeLa cells and Vero cells were purchased directly from ATCC. All control SV40-fibroblast cell lines, as well as control hES or iPS cell lines used in our experiments to compare with TMEFF1-mutated cells, have been sequencing confirmed to be wild type for TMEFF1. All hES or iPS cell lines were karyotyped to ensure that the genome was intact.

Mycoplasma contamination

All cells were regularly checked to ensure that they are mycoplasma negative.

Commonly misidentified lines
(See ICLAC register)

No commonly misidentified lines were used.

Plants

Seed stocks

Not applicable as no plants are involved.

Novel plant genotypes

Not applicable as no plants are involved.

Authentication

Not applicable as no plants are involved.

Flow Cytometry

Plots

Confirm that:

☒ The axis labels state the marker and fluorochrome used (e.g. CD4-FITC).

☒ The axis scales are clearly visible. Include numbers along axes only for bottom left plot of group (a 'group' is an analysis of identical markers).

☒ All plots are contour plots with outliers or pseudocolor plots.

☒ A numerical value for number of cells or percentage (with statistics) is provided.

Methodology

Sample preparation

HEK293T cells were transfected with empty vector or plasmid containing WT or various patient-specific mutant TMEFF1 sequences. Cells were then either permeabilized or not permeabilized, and stained with anti-TMEFF1 antibody. Cells were then acquired with Beckman Coulter Gallios.

HEK293T cells were treated with recombinant His-tagged HSV-1 gD, or infected with HSV-1 and harvested at various timepoints. Cells were then either permeabilized or not permeabilized, and stained with PE-conjugated anti-NECTIN-1 antibody or APC-conjugated anti-His antibody. Cells were then acquired with BD LSRII flow cytometer.

Instrument	Beckman Coulter Gallios and BD LSRII flow cytometer
Software	FlowJo software (V10.9.0, Tree Star)
Cell population abundance	At least 5000 cells were acquired per sample for analyses
Gating strategy	Gating was based on isotype control stain, cells that were knockout of marker of interest, or cells that were untreated.

☒ Tick this box to confirm that a figure exemplifying the gating strategy is provided in the Supplementary Information.
